# TLR7/8/9 agonists and low-dose cisplatin synergistically promotes tertiary lymphatic structure formation and antitumor immunity

**DOI:** 10.1038/s41541-024-01055-z

**Published:** 2025-01-19

**Authors:** Shuting Wu, Rong Xiang, Yiwei Zhong, Shushu Zhao, Zhiyu Zhang, Zhihua Kou, Shijie Zhang, Yi Zhao, Cheng Zu, Gan Zhao, Yanling Xiao, Sulin Ren, Xiaoming Gao, Bin Wang

**Affiliations:** 1 Key Laboratory of Medical Molecular Virology, School of Basic Medical Sciences, Fudan University, Shanghai, 200032 China; 2 Shanghai Institute of Infectious Disease and Biosecurity, Fudan University, Shanghai, 200032 China; 3 Advaccine Biopharmaceutics (Suzhou) Co. LTD, Suzhou, 215000 China; 4 Precision Scientific (Beijing) LTD., Beijing, 100085 China; 5 Present Address: The Wistar Institute, Philadelphia, 3601 Spruce Street, PA 19104 USA

**Keywords:** Cancer immunotherapy, Adjuvants

## Abstract

In situ vaccination (ISV) triggers antitumor immune responses using the patient’s own cancer antigens, yet limited neoantigen release hampers its efficacy. Our novel combination therapy involves low-dose local cisplatin followed by ISV with a TLR7/8/9 agonist formulation (CR108), in which CR108 boosts and sustains the antitumor responses induced by the cisplatin-released neoantigens. In mouse models, the cisplatin+CR108 combination significantly outperformed cisplatin or CR108 alone in abrogating established 4T1 and B16 tumors. The synergistic antitumor effects of cisplatin and CR108 were accompanied by markedly increased tumor tertiary lymphatic structures (TLS) formation, higher levels of type I and III interferons and TNF-α in serum, augmented T and B lymphocyte infiltration, antigen-presenting cell activation, as well as reduced functionally of exhausted T cells. Single-cell sequencing analysis uncovered a potential pathway for TLS to serve as a reservoir for functional antitumor effector T cells. Furthermore, cisplatin+CR108 combo therapy, but neither cisplatin nor CR108 alone, effectively inhibited the growth of treated 4T-1 tumor in an effector T cell-dependent manner. Notably, the combo therapy also suppressed the growth of distant untreated 4T-1 tumors, demonstrating systemic antitumor effects. Moreover, combo-therapy led to full regression of 4T-1 tumors in a large percentage of mice, who became strongly resistant to secondary tumor challenge, a clear indication of antitumor immunological memory. The cisplatin+CR108 combo therapy holds promise in converting “cold” tumors into “hot” ones and eliciting robust antitumor immune responses in vivo.

## Introduction

While chemotherapeutic drugs such as cisplatin remain the first-line choice in most cancer therapies, they are rarely curative due to the development of drug resistance and severe adverse side effects^1^. Recently, groundbreaking developments in tumor immunotherapy have yielded remarkable clinical achievements through the activation of cytotoxic T lymphocytes (CTLs) within the immune system^2,3^. CAR-T cell and immune checkpoint (ICP) blockade therapies exhibit promising antitumor effects, yet their effectiveness is limited to a small percentage of patients^4–6^. Advances in neoantigen identification technology have reignited interest in “personalized vaccination therapies”. However, these anticancer vaccines have shown disappointment, evident in the low overall objective response rates observed in several clinical trials^7^.

In situ vaccine (ISV) remains a promising cancer immunotherapy strategy that consists of intratumoral/peritumoral administration of immunostimulatory molecules (vaccine adjuvants) represented by toll-like receptor (TLR) agonists^8–10^. The rationale is that intratumoral/peritumoral injection of proinflammatory adjuvants helps to turn the immunosuppressive (cold) tumor microenvironment (TME) into an immunologically active (hot) one. As opposed to traditional vaccines, where antigens are carefully selected, purified, or prepared and injected into patients, the in situ approach utilizes the neoantigens in TME itself by sourcing the antigens from dead or dying tumor cells^11^. One of the commonly used adjuvants for ISV is unmethylated cytosine-phosphorothioate-guanine containing oligodeoxynucleotides (CpG), a TLR9 agonist that functions to stimulate the host immune response through plasmacytoid dendritic cells (pDC), monocytes, and nature killer (NK) cells^10^. Additionally, CpG itself has mild antitumor activity and has been used previously in the treatment of early-stage malignant melanoma^9^. Resiquimod (R848), an immunomodulating imidazoquinoline derivative, is a specific ligand for human TLR7/8 or murine TLR7 expressed on DCs, macrophages, NK, and B cells that are capable of enhancing anti-viral and antitumor humoral and Th1-type cellular immune responses in vivo^12^. When incorporated into nanoparticles, R848 was effective as an adjuvant in animal models of ISV^13^.

Despite much progress in ISV studies, the antitumor efficacy of ISV therapy is limited by the insufficient release of neoantigens. To overcome this problem, we designed a combination therapy consisting of peritumoral administration of low-dose cisplatin followed by a TLR7/8/9 agonist formulation (CR108, consisting mainly of CpG1080 and R848) as an ISV adjuvant. By cross-linking DNA and inhibiting mitosis, cisplatin can cause immunogenic apoptosis of cancer cells and the release of damage-associated pattern molecules (DAMPs) as well as tumor-associated antigens (TAAs)^14^. The cisplatin-unleashed neoantigens from the patient’s own tumor could be captured by DCs and presented to T cells^15^. Additionally, cisplatin can promote immune recognition and immune-mediated tumor elimination in a mouse model of melanoma^14^. It is thus likely that pretreatment with cisplatin could significantly boost the efficacy of ISV. We herein report that, in the mouse models of 4T1 and B16 solid tumors, cisplatin and CR108 combination therapy achieved synergistic effects with better efficacy, accompanied by cold-to-hot tumor conversion and systemic and long-term antitumor adaptive immune responses. Our data support the concept that the cisplatin+ISV combination might be a valuable strategy in anticancer immunotherapy.

## Results

### Synergistic antitumor effects of cisplatin and CR108 combo therapy in immunocompetent mice

**Fig. 1:**
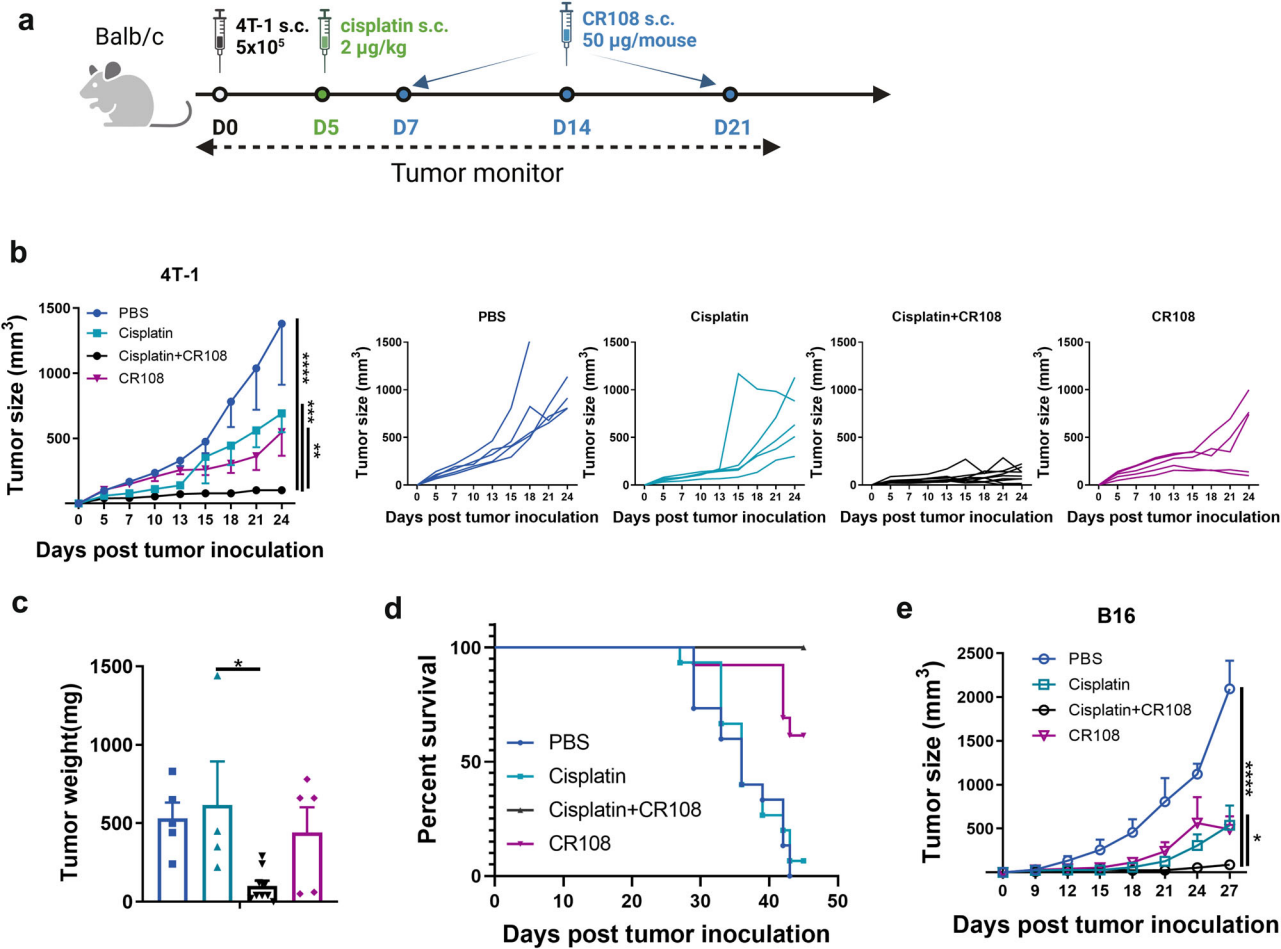
Enhanced antitumor efficacy of the cisplatin + CR108 combo therapy. **a** The design of the unilateral 4T1 tumor model against primary tumors is depicted schematically. BALB/c mice (cisplatin+CR108, *n* = 9; others, *n* = 5) were implanted with 5 × 10^5^ viable 4T1 cells (solid black dot) s.c. in right flanks, and tumors to reach a palpable size of approximately in 4–5-mm diameter was peritumorally administered with cisplatin (solid green dot) followed by three doses of CR108 (with 7-day intervals, solid blue dot). Mice receiving either PBS, cisplatin, or CR108 alone were included as controls. Tumor growth was monitored over a period of 25 days. The image was created with www.Biorender.com and was licensed. **b** Average primary tumor growth curves and individual tumor growth size curves after various treatments were shown on the 4T1 model, with left panel displaying average size and the right panels showing individual responses. **c** Tumor weight was monitored at 24DPI endpoint. **d** Separate groups were monitored for survival rates of 4T-1 tumor-bearing mice following various treatments described in (**a**); *n* = 15 for PBS or cisplatin groups, *n* = 13 for cisplatin+CR108 or CR108 groups. **e** C57BL6 mice received the same treatment scheduled as outlined in (**a**) for the unilateral B16 tumor model, replacing the BALB/c mice with C57BL6 mice (*n* = 6 per group). The mice were implanted with 3 × 10^5^ viable B16F10 cells s.c. in right flanks. Mice receiving either PBS, cisplatin, or CR108 alone were included as controls. Average primary tumor growth curves were depicted on the B16 model. Statistics significance were determine by two-way ANOVA and one-way ANOVA tests. Error bars represent mean ± SEM. *P < 0.05, **P < 0.01, ***P < 0.001, ****P < 0.0001.

Cisplatin+CR108 combo therapy was compared with cisplatin or CR108 monotherapies for the ability to inhibit the growth of s.c. implanted 4T1 and B16 tumors in immunocompetent syngeneic mice (Fig. 1a). Based upon our earlier dosage optimization results (Supplemental Fig. 1), cisplatin and CR108 were used at 2 μg/kg and 50 μg/mouse, respectively. Mice of BALB/c and C57BL/6 strains were s.c. engrafted on the right flank with 5 × 10^5^ 4T1 breast cancer cells or 3 × 10^5^ B16F10 melanoma cells, respectively. When the engrafted tumors reached approximately 5 mm in diameter, cisplatin, or PBS as control, was peritumoral injected, followed by three injections of CR108, or PBS, at weekly intervals (Fig. 1a). The growth of the implanted tumors in the treated animals was monitored for up to 27 days post-inoculation (DPI). As shown in Fig. 1b, c, cisplatin + CR108 combo treatment almost completely abrogated the implanted 4T1 tumor, while cisplatin or CR108 monotherapy exhibited little or only marginal therapeutic effects. Furthermore, cisplatin+CR108 combo therapy significantly prolonged the survival of the tumor-bearing mice (Fig. 1d). Moreover, the cisplatin+CR108 combo therapy exhibited a similarly potent antitumor effect in mouse melanoma (B16) model (Fig. 1e). These data provide strong evidence for synergistic effects of cisplatin+ CR108 combo therapy against established solid tumors in vivo.

### Cisplatin + CR108 combo therapy facilitates TLS formation and maturation

**Fig. 2:**
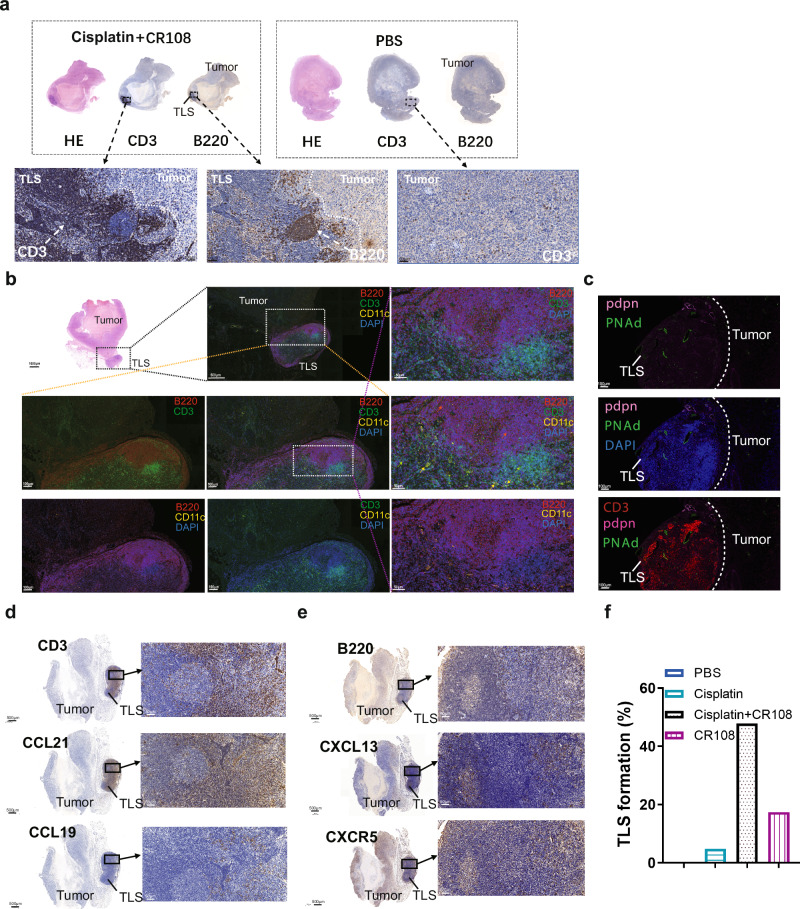
Effects of cisplatin + CR108 combo therapy on the formation of TLS. **a** Immunohistochemical analysis revealed the formation of TLS with 4T1 tumor cells on 28DPI followed the same treatment scheduled as outlined in Fig. 1a. **b** Immunofluorescence revealed the structure and distribution of T, B cells, and DCs on 28DPI in TLS. **c** Immunofluorescence revealed the structure and distribution of PDPN, PNAd, and CD3^+^T cells on 21DPI. **d** Immunohistochemical analysis revealed the expression of CCL19, CCL21, and CD3 of TLS on 21DPI. **e** Immunohistochemical analysis revealed the expression of CXCL13, CXCR5, and B220 of TLS on 21DPI. **f** The bar graph presented the proportions of TLS formation across various treatment groups. The cisplatin treatment group had a sample size of 21, while the other groups had 23 samples for each. The TLS formation percentage was calculated by dividing the number of TLS formation samples by the total number of samples in each group, and then multiplied by 100%.

TLSs are ectopic lymphoid-like formations that can develop within the TME or in other non-lymphoid tissues^16,17^. Recent evidence suggests that TLS development and maturation in TME are associated with stronger antitumor immunity and a better prognosis^18–20^. The TLSs were found both inside and external to the tumors in cisplatin+CR108 combo-treated mice. The TLS nodules consisted mainly of B (B220^+^) and T (CD3^+^) lymphocytes, as evidenced by immunohistochemical examination (Fig. 2a and Supplementary Figs. 2, 3a–c). The observation via digital slide scanner of the TLSs further identified a follicle-like structure consisting of large T cell-rich zones with scattered CD11c^+^ DCs surrounded by patches of B220^+^ B cell aggregates (Fig. 2b and Supplementary Fig. 3d–f). The structure is similar to the “TLS germinal centers” reported by previous investigators ^20–22^.

Podoplanin (PDPN), a transmembrane receptor glycoprotein upregulated in cancer-associated fibroblasts and inflammatory macrophages, contributes to the formation and maturation of TLSs^23^. Interestingly, PDPN expression was detected at TLS edges (Fig. 2c). High endothelial venules (HEVs), characterized by elevated PNAd expression, were also observed in the T cell-rich zone of TLSs of the cisplatin+CR108 combo-treated tumors (Fig. 2c). Further immunohistochemical analysis revealed the presence of CCL21 and CCL19 in the T cell zones of TLSs (Fig. 2d). Moreover, a large quantity of CCL21, a HEV-secreted chemokine related to B and T cell recruitment into TME^24^, was found to be present in TLS-adjacent tumor tissues. Another signature chemokine (CXCL13) and its receptor (CXCR5) were detected in B cell-rich zones of tumor/TLS sections (Fig. 2e). Following a series of meticulous observational studies and data analysis on the regressed and control tumors, TLSs external to tumors became discernible to the naked eye upon dissecting the mice (Supplementary Fig. 3g). This facilitated a straightforward determination of the percentage of TLS formation within both the treated and untreated groups. Notably, external TLSs were found in nearly half of the cisplatin+CR108 combo-treated 4T1 solid tumors, while less than 20% of the cisplatin- or CR108-treated 4T1 tumors were positive, and none were detected in the PBS-treated mice (Fig. 2f). These data provide strong evidence for cisplatin+CR108 combo therapy to facilitate TLS formation and maturation in TME, which could be a crucial event related to its antitumor efficacy.

### Analysis of TLS-resident cells in cisplatin + CR108 combo-treated tumors

**Fig. 3:**
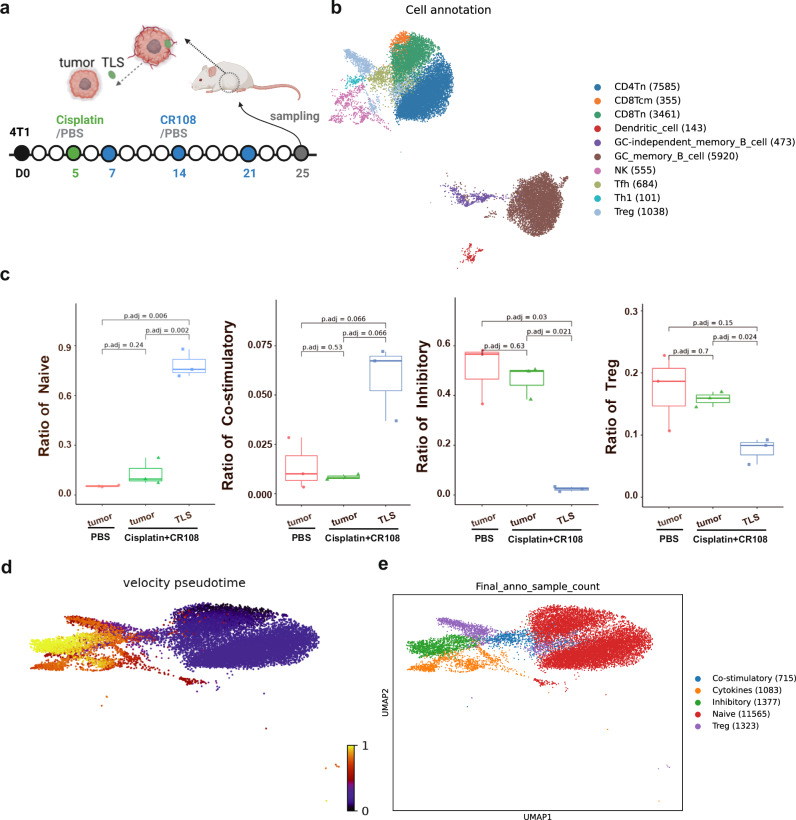
Analysis of T and B cells in tumor and TLS revealed by scRNA-seq. **a** The design of collecting 4T1 tumors and TLS is depicted schematically. BALB/c mice (*n* = 3 per group) were implanted s.c. with 5 × 10^5^ viable 4T1 cells in right flanks, and tumors to reach a palpable size of approximately 5 mm in diameter were peritumorally administered with cisplatin followed by three doses of CR108 in 7-day intervals. Mice received PBS-treated alone were used as the negative control. The cisplatin + CR108 combo-treated 4T1 tumors were surgically separated into TLS nodules (labeled as “TLS”) and external TLS-depleted tumor themselves (labeled as “Tumor”); whereas tumors (labeled as control Tumor) from the PBS group were used as controls. All samples were collected 25 DPI to prepare for next-generation sequencing (NGS), respectively. The image was created with www.Biorender.com and was licensed. **b** t-distributed stochastic neighbor embedding (t-SNE) plot of immune cells in TLS, with cell counts indicated in parentheses. **c** The ratio of naïve, costimulatory, Treg, and inhibitory T cells was plotted against each treated group. **d** RNA velocity analysis for T and NK cells, with the color-coded for pseudotime. **e** t-SNE plot of T and NK cells, with each color representing one of the subgroups in TIL and TLS. Statistics: The Wilcoxon rank-sum test was performed to compare ratios of each kind of T cell in different treatment groups and resulted in *P* values were corrected for multiple testing via the method of Benjamini & Hochberg.

To elucidate potential differences in immune cell composition and function between tumors treated with the combo therapy or PBS, 4T1 tumors were surgically dissected into two fractions: external TLS (designated as “TLS”) and external TLS-depleted tumor (designated as “Tumor”) for single-cell RNA sequencing (Fig. 3a). Samples of PBS-treated tumors served as a control and designated as “control tumor”. After integrating and quality controlling the transcriptome data, we annotated all cell types, including T cells, tumor-originating cells, endothelial cells, fibroblasts, B cells, granulocytes, monocytes, macrophages, dendritic cells, binucleated cells, cells with high proportion expression of mitochondrial genes and rare islet cells (Supplementary Fig. 4a and Supplementary Table 2b).

Unsupervised clustering revealed ten distinct populations among TLS-resident immune cells, including diverse T cell subsets like CD4/8 naïve (Tn), CD8 central memory (Tcm), Th1, Tfh, and Tregs, alongside DCs, NK cells, and germinal center (GC) memory B cells (Fig. 3b and Supplementary Fig. 4b). While similar clusters were present in TILs from the external TLS-depleted tumors and tumors of the PBS-treated control group, their frequencies differed dramatically (data not shown). Notably, TLSs harbored significantly higher proportions of Tn and costimulatory T cells compared to TILs, with a concomitant decrease in Tregs and inhibitory T cells (Fig. 3c and Supplementary Fig. 4c). Interestingly, the distribution of T cell clusters in TILs of the external TLS-depleted tumors mirrored that of the PBS-treated control group. This skewed distribution of “costimulatory” versus “inhibitory” T cells aligns with the concept of TLS-driven cold-to-hot tumor conversion.

To unravel the potential developmental trajectory of T cells within TLSs, RNA velocity analysis was carried out based on the fitting of a steady-state ratio of unspliced and spliced mRNA counts for each gene. The velocity pseudotime, i.e., the random walk-based distance measurement on the velocity graph, was calculated and displayed in Supplementary Fig. 4d and Fig. 3d, e show that Tn cells in TLSs were mainly located at the beginning of the pseudotime trajectory, whereas inhibitory T cells appeared primarily at the end, implying a potential development pathway in which Tn cells were converted into costimulatory T cells, followed by sequential effector phases, and finally exhaustion. The ratio of transcriptions from the naïve and costimulatory T cells were initiated earlier and significantly higher but significantly lower for the inhibitory and Treg cells in the TLS group, whereas the significantly higher proportion of inhibitory and Treg cells and lower of naïve and costimulatory T cells were seen in the PBS-treated and TLS-minus tumor groups (Supplementary Fig. 4e). This finding suggests that TLS-resident Tn cells might serve as a reservoir for effector T cells within the tumor.

To further explore intercellular interactions within mature TLSs, we performed cell-cell communication analysis, which revealed robust and clear interactions between GC memory B cells and both CD8^+^ and CD4^+^ Tn cells (Supplementary Fig. 5a–c). This suggests a crucial role of GC memory B cells in antigen presentation within TLSs, contributing to the potent antitumor response observed.

In conclusion, the scRNA sequencing analysis provides compelling evidence for the unique composition and potential developmental pathways of T cells within TLSs generated by cisplatin+CR108 combo therapy. The high abundance of costimulatory T cells and their potential derivation from Tn cells point to TLS as a reservoir for antitumor effector T cells. Additionally, the robust interactions between GC memory B cells and Tn cells highlight their collaborative role in antigen presentation within TLSs, further contributing to the observed antitumor efficacy. These findings shed light on the intricate cellular mechanisms underlying the success of cisplatin+CR108 combo therapy in driving cold-to-hot tumor conversion and potentially pave the way for future therapeutic interventions targeting TLSs.

### Boosting immune infiltration and function with cisplatin + CR108 combo therapy

**Fig. 4:**
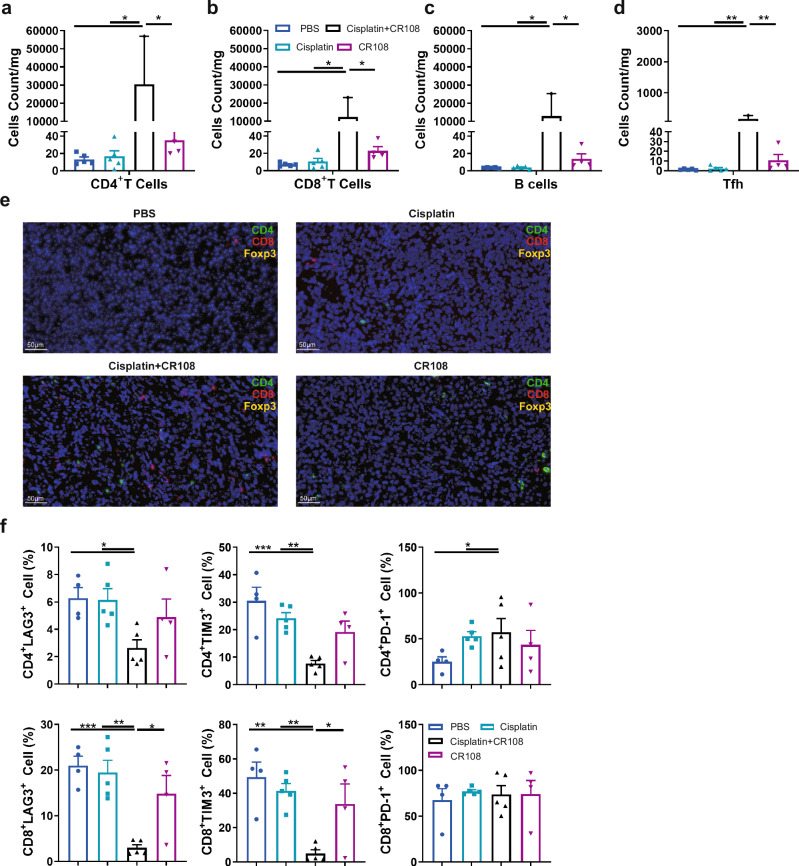
Effects of cisplatin + CR108 combo therapy on TILs. **a**–**d** Quantification of CD4^+^T (**a**), CD8^+^T (**b**), B220^+^B (**c**), and PD-1^+^CXCR5^+^Tfh (**d**) cells infiltrating into the tumor in 28DPI were analyzed by FACS. There were five mice per group, and the tumor tissue samples diverged due to the fact that the tumor size in the treatment group is insufficient to obtain single-cell suspension. Other group tumors, *n* = 5; CR108 tumors, *n* = 4; cisplatin+CR108 tumors, *n* = 2. **e** Tumor sections were stained with immunofluorescent labeled specific antibodies and detected the presence of infiltrated CD4^+^T, CD8^+^T, and Treg cells. **f** The expression of exhaustion factors of tumor-infiltrated CD4^+^T cells and CD8^+^T cells 28DPI were used to analyze by FACS. *n* = 5, per group. Statistics significance was determined by one-way ANOVA for (**a**–**d**) and (**f**). Error bars represent mean ± SEM. *P < 0.05, **P < 0.01, ***P < 0.001, ****P < 0.0001.

Immune cell infiltration plays a pivotal role in cancer prognosis, distinguishing “hot” from “cold” tumors. Hot tumors are characterized by high T cell infiltration and often respond well to immunotherapy^25^. To unravel the immune landscape of cisplatin+CR108 combo-treated tumors, we analyzed 4T1 tissues from BALB/c mice 28 DPI using flow cytometry and immunofluorescence techniques. Compared to PBS and cisplatin alone, CR108 treatment modestly increased tumor-infiltrating lymphocytes (TILs). However, cisplatin+CR108 combo therapy led to a 100-fold surge in CD4^+^ Th, Tfh (PD-1^+^CXCR5^+^), CD8^+^ CTL, and B220^+^ B cells (Fig. 4a–d and Supplementary Fig. 6a–d). Immunofluorescence staining further corroborated the dramatic influx of CD4^+^ T, CD8^+^ T and Tregs cells into the tumor tissue (Fig. 4e and Supplementary Fig. 6e). Quantitative analysis on CD4^+^, CD8^+^, and CD4^+^Foxp3^+^ populations in the IF-stained specimens confirmed the significant increase in infiltrating T cells in the cisplatin+CR108 group compared to other treatment groups (Supplementary Fig. 6f). Depleting either CD4^+^ or CD8^+^ T cells prior to treatment significantly compromised survival (Supplementary Fig. 7a–c), highlighting their critical role in the antitumor response.

LAG-3, TIM-3, and PD-1 are ICPs that can limit T cell function^26^. Flow cytometric analysis revealed a significant decrease in LAG-3^+^ and TIM-3^+^ (but not PD-1^+^) CD4^+^ and CD8^+^ TILs in the cisplatin+CR108 group compared to other treatments (Fig. 4f and Supplementary Fig. 8a). Furthermore, the cisplatin+CR108 combo-treated group exhibited a significant decrease in double positive expressions of TIM-3 and PD-1, or LAG-3 and PD-1, and triple-positive of TIM-3, PD-1, and LAG-3 on CD4^+^ and CD8^+^ TILs compared to other treatments (Supplementary Fig. 8b–h). This suggests that the cisplatin+CR108 combo therapy was significantly more efficient than monotherapies in boosting tumor infiltration by functional T and B cells, coupled with reduced T cell exhaustion.

### Unleashing systemic antitumor immune response: cisplatin + CR108 boosts cytokines, DCs, and T Cells

**Fig. 5:**
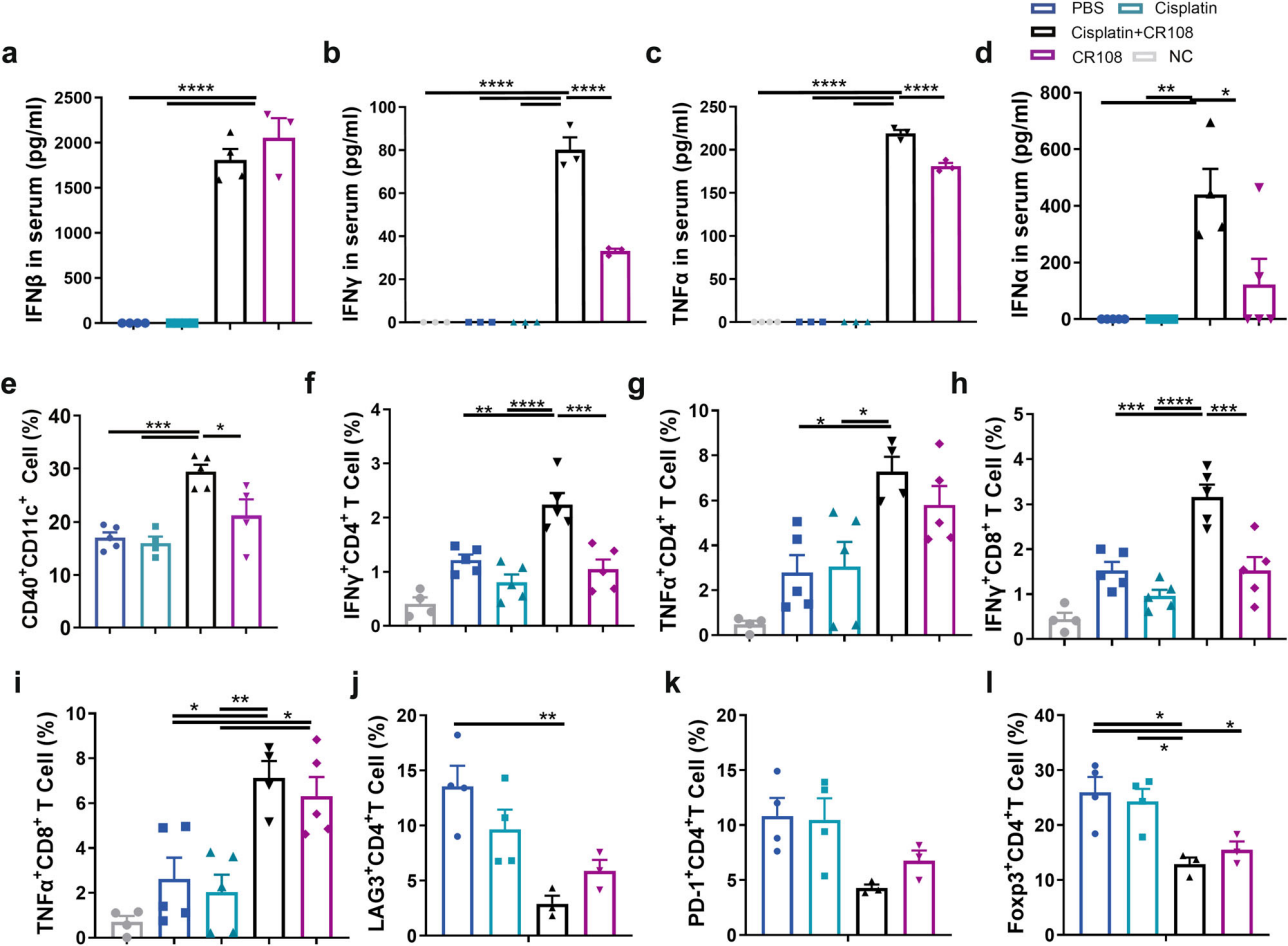
Effect of cisplatin + CR108 combo therapy on the activation and maturation of APCs and T cells in dLN. Mice inoculated with 5 × 10^5^ viable 4T1 cells s.c. and subsequently treated with various regimens were performed according to the schedule described in Fig. 1a. The serum levels of IFNβ (**a**), IFNγ (**b**), and TNFα (**c**) were measured four hours after the first dose of CR108 treatment (7DPI). **d** The serum levels of IFNα were analyzed 4 h after the second dose of CR108 treatment (14DPI), *n* = 5 per group. **e** Costimulatory signal expression of CD40 in DCs (CD11c^+^CD11b^+^) from the dLN was performed on 9 DPI. cisplatin, *n* = 4; CR108, *n* = 4; others, *n* = 5. **f**–**i** Frequency of IFNγ and TNFα in CD4 and CD8 T cells were analyzed by flow cytometry. Lymphocytes at 2 × 10^6^ isolated 17 DPI from the dLN were stimulated in vitro with 5 ng of PMA and 50 ng of Ionomycin for 4 h and analyzed by a flow cytometry gated on CD4^+^ cells (**f**, **g**) or on CD8^+^ T cells (**h**, **i**). NC, *n* = 4; others, *n* = 5. **j**–**l** Expression of exhaustion factors, LAG-3 or PD-1 (**j**, **k**) in CD4^+^T cells, and frequency of Treg (Foxp3^+^CD4^+^) (**l**) in dLN 28 DPI were assessed by flow cytometry. cisplatin+CR108, *n* = 3; others, *n* = 4. Statistics significance was determined by one-way ANOVA. Error bars represent mean ± SEM. *P < 0.05, **P < 0.01, ***P < 0.001, ****P < 0.0001.

The potent antitumor synergy of cisplatin+CR108 combo therapy suggests a robust systemic immune response. To investigate this, we focused on several key players in antitumor immunity: interferons (IFNα, IFNβ, IFNγ), TNF-α, DCs, and T lymphocytes. As expected, CR108 alone triggered sharp increases in serum IFNα-β-γ and TNF-α levels. Notably, the cisplatin+CR108 combo dramatically amplified this effect for IFNα, IFNγ, and TNF-α, showcasing a potent synergistic activation of cytokine production pathways by the combined therapy. This synergy was not observed for IFNβ (Fig. 5a–d).

Activation of the TLR pathway in DCs plays a crucial role in DC maturation and activation. Since the CR108 contains CpG, a TLR9 agonist, and R848, a TLR7/8 agonist, the treatment with CR108 should trigger TLR activation. As depicted in Supplementary Fig. 9a, cisplatin+CR108 combo treatment increased expressions of the *Tlr7* gene in DCs in TLS, *Tlr9* in DCs in tumors compared to the PBS-treated group. Simultaneously, cisplatin+CR108 combo therapy led to a significant increase in the percentages of activated (CD40^+^) CD11c^+^DCs (both CD11b^+^ and CD11b^−^) in dLN compared with other groups (Fig. 5e and Supplementary Fig. 9b, c). The results indicate that TLR agonists in the combo regimens could activate the TLR signal pathways and further enhance DC maturation and migration to dLNs.

We next analyzed the functional status of T lymphocytes in dLNs. cisplatin+CR108 combo treatments significantly boosted both IFNγ and TNFα expressing CD8^+^ and CD4^+^ T cells in dLN of 17 DPI mice compared to that of the PBS or cisplatin groups (Fig. 5f–i and Supplementary Fig. 9d, e). Interestingly, CR108 by itself had little to no effect on these cytokines, except for TNFα^+^CD8^+^ T cells.

Furthermore, the levels of CD4^+^ T cells expressing Foxp3^+^ (Tregs), LAG-3, or PD-1 were significantly reduced in the dLN of mice with cisplatin+CR108 or CR108, albeit CR108 was less effective than cisplatin+CR108 (Fig. 5j–l). Taken together, three lines of evidence endorse systemic antitumor immune activation following cisplatin+CR108 combo therapy, including (i) elevated serum levels of IFN-Is and TNFα; (ii) increased DC maturation and dLN migration; (iii) T cell activation-suppression balance in dLNs in favor of the former. This coordinated immune response aligns with the dynamic T cell development observed in the TME after cisplatin+CR108 combo treatment (Fig. 3d, e), further solidifying the potent synergy of this combination therapy.

### Cisplatin + CR108 combo-therapy triggers abscopal effects

**Fig. 6:**
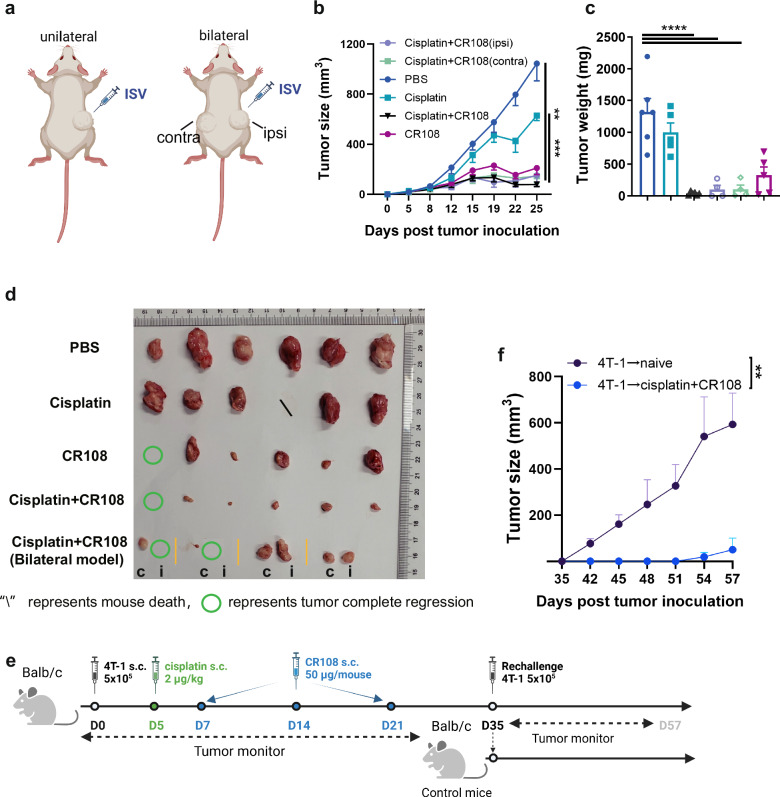
Tumor inhibition of cisplatin + CR108 combo therapy in a two-tumor and rechallenge mouse model. **a** The design of the unilateral 4T1 tumor model (left panel) and bilateral 4T1 tumor model (right panel) against primary tumor challenges is depicted schematically. The therapeutic treatments were adopted in (Fig. 1a). In the unilateral 4T1 tumor model, tumor cells at 5×10^5^ to reach a palpable size of approximately 4–5 mm in diameter were peritumorally administered with cisplatin followed by three doses of CR108 in 7-day intervals, whereas additional tumor cells were implanted into contralateral side of mice and treated as above in the bilateral model. Tumor growth was monitored on both sides over a period of 25 days. The “ipsi, or i” represents the ipsilateral side of the administration, and the “contra, or c” represents the contralateral side of the administration. The image was created with www.Biorender.com and was licensed. **b** Primary tumor growth curves on average. bilateral 4 T1 tumor, *n* = 4; others, *n* = 6. **c** Tumor weight at the tumor monitor’s endpoint. bilateral 4 T1 tumor, *n* = 4; others, *n* = 6. **d** Separated tumors from all groups were photographic recorded at the termination of the study on 25 DPI. **e** Schematic diagram illustrating the design of the rechallenge 4T-1 tumor model. 4T-1 tumor-bearing mice (*n* = 3) were combo treated with cisplatin + CR108 according to the schedule showed in Fig. 1a. Naïve mice (*n* = 5) served as controls. After the initial treatment and elimination of the primary tumors (35DPI), the mice were rechallenged with the same type of tumor cells in the same flank. Tumor growth was monitored and measured at regular intervals. **f** Rechallenge tumor growth curves. Statistics significance was determined by two-way ANOVA, and one-way ANOVA tests were used to determine statistical significance. Error bars represent mean ± SEM. *P < 0.05, **P < 0.01, ***P < 0.001, ****P < 0.0001.

Given the potent systemic antitumor immune responses induced by cisplatin+CR108 combo therapy in mice bearing solid tumors, an “abscopal effect” – tumor regression at distant untreated sites, would be expected. To test this hypothesis, we employed a two-tumor mouse model where 4T1 cells were s.c. implanted at two separate locations (Fig. 6a). Once tumors reached a palpable size (4–5 mm), cisplatin+CR108 combo treatment was administered to only one tumor following the established protocol. Remarkably, tumor growth was significantly inhibited not only in the treated (ipsilateral, ipsil) but also in the distant untreated (contralateral, contra) tumors (Fig. 6b–d). To further address the question of whether the cisplatin+CR108 combo-treated tumor-bearing mice would gain long-term antitumor immunity, the fully recovered mice who remained tumor-free for 2 weeks were rechallenged with freshly implanted 4T-1 tumors. As shown in Fig. 6e, f, these animals were strongly resistant to the secondary tumor challenge, a clear indication of long-term antitumor immunological memory. This striking finding strongly aligns with the systemic immunological response induced by the combination therapy, demonstrating its potential to combat cancer beyond the initial site of intervention. Collectively, these findings suggest that the cisplatin+CR108 combination treatment may have a strong potential to facilitate long-term tumor control and prevention of relapse.

## Discussion

Conventional ISV has faced limitations against aggressive “cold” tumors like melanoma and triple-negative breast cancer (TNBC)^27,28^. Our study presents a novel strategy that not only surpasses these limitations but unlocks a key mechanism in tumor rejection—the emergence of organized immune hubs within the TME known as TLSs^18,19^. In this strategy, peritumoral low-dose cisplatin is administered to kill tumor cells (see Supplementary Fig. 1h). This treatment is followed by the administration of CR108, a potent TLR7/8/9 agonist, which is expected to stimulate APCs (Fig. 5e) and subsequently enhance host antitumor immunity (Fig. 1). This synergy hinges on two crucial factors. 1) Robust Immune Infiltration: cisplatin+CR108 combo treatment goes beyond simply attracting immune cells to the TME. It orchestrates a shift towards activated cDC1s, reduces inhibitory T cells, and fosters a potent Th1/Tc1 response, thereby creating a microenvironment primed for coordinated antitumor activity; 2) TLS Formation: Unlike the “immune-excluded” control tumors and the “immune-infiltrated” CR108-treated ones, the cisplatin+CR108 combo-treated tumors are more likely to develop organized TLSs. These mature structures, rich in GCs (Supplementary Fig. 3b, c), provide a dedicated platform for antigen presentation, antibody production, and immune cell interaction, which is clinically associated with a good prognosis^19,29^. RNA rate analysis suggests that naïve T cells in TLS can be activated as functional T cells. In our opinion, TLSs function as a “training camp” for the preparation/recruitment of anticancer rookies, adjunction to the “battlefield” for direct fighting between the cancer cells and activated lymphocytes. It is perhaps not surprising that the TLS-residing lymphocytes remained naïve till the end of the experiment, they would only become “armed soldiers” when infiltrating into cancer tissues. Although this is still a conjecture requiring further experimental confirmation, all current experimental data indicate that TLS formation is associated with a good prognosis and increased survival. This organized architecture surpasses the diffuse infiltration seen in other ISV approaches, leading to sustained and potent antitumor activity.

Molecular mechanisms for the formation and biological function of tumor-associated TLSs is a focus of current cancer research^30^. In our study, combo-therapy appeared to be significantly more effective than cisplatin or CR108 monotherapies in driving TLS formation (Fig. 2f). This is likely attributable to the synergy between the tumoricidal effect of cisplatin and local immune inflammation induced by TLR agonists. Cisplatin-released tumor DAMPs and neoantigens may create a nitch for APC activation, while TLR agonists are known to trigger the production of inflammatory cytokines and chemokines. Our preliminary analysis of the scRNA-seq data revealed a higher level of LtB expression in the TLS-residing cells compared to TILs of the external TLS-removed tumors (data not shown). LT-α/LT-β can promote the expression of chemokines CCL19, CCL21, and CXCL13, which help recruit lymphoid tissue-induced cells (LTi) to lesion sites. More detailed research will be needed to provide further mechanistic insights regarding how the combination of cisplatin and TLR agonists induces TLS in a more efficient manner.

Cisplatin is an effective chemotherapeutic drug, and its clinical application is often limited by severe ototoxicity, nephrotoxicity, and other side effects associated with high-dose treatment^31,32^. In the present study, however, cisplatin was used not for its traditional chemotherapeutic potential. Instead, it was included as part of a novel ISV therapy, which only requires a small amount of cisplatin administered via peritumoral rather than systemic (i.v. or i.p.) route. The aim of low-dose (2 μg/kg) cisplatin peritumoral injection was to drive tumor antigen release from cancer cells, which was verified by tumor cell apoptosis within 24 h after cisplatin administration in vivo using TUNEL immunofluorescence staining (as shown in Supplementary Fig. 1h). Tumor antigens were likely recognized and picked-up by APCs, which could then present the tumor-derived antigens to host T cells, potentially triggering an immune response against the remaining cancer cells.

A wide range of TLR-agonist agents have been tested to date in preclinical and clinical ISV studies. The US Food and Drug Administration (FDA) has approved the tuberculosis BCG vaccine as an ISV treatment for superficial bladder carcinoma and R848 for skin carcinomas^33,34^. Sagiv-Barfi et al. recently documented that intratumoral CpG injection induced the expression of OX40 on CD4^+^ T cells in mouse or human tumors, triggering a systemic immune response^35^. TLR9 agonists CMP-001 and IMO-2125 have been studied in combination with systemic immune checkpoint blocking therapy, and the ongoing clinical trials recently reported promising interim results^36^. There have also been studies on STING-based ISV therapies against cancers, albeit the role of STING signaling in antitumor immunity seems controversial^37,38^. Given that CR108 therapy benefits greatly from peritumoral pre-administration of low-dose cisplatin, a combination of additional chemotherapeutic drugs and immune adjuvants would be worthy of testing.

The 4T1 and B16 tumors of the control groups that had been treated with peritumoral PBS (or cisplatin) in the absence of CR108 appeared to be “immune-excluded” because only limited numbers of tumor-infiltrating immune cells were observed. On the other hand, the CR108-treated tumors were “immune-infiltrated”, as they contained CD4^+^ and CD8^+^ cells expressing checkpoint molecules such as PD-1 and CTLA4. This, however, was incomparable to the hot tumors of the cisplatin+CR108 combo-treated group, in which not only heavy immune infiltration but also the development of TLSs was observed. Although there are distinct T and B cell zones in TLS (Fig. 2b and Supplementary Fig. 3d–f), significant signal connections were observed between GC memory B cells and T cells via Class I and II MHC molecules (Supplementary Fig. 5c). Such intercellular communications could play a pivotal role in the antitumor immune responses. Chemoattractant CXCL13 was found expressed in cisplatin + CR108 combo-treated tumors, which could recruit B and T cells to the B cell zone or follicles in TME. Such an immunological phenomenon has recently been linked with improved survival in cancer ^19,21,39,40^.

Based on the evidence accumulated so far, molecular mechanisms underlying the cisplatin+CR108 combo therapy against cold tumors are hypothesized. First, the peritumoral injected low-dose cisplatin may release immunogenic neoantigens from the tumor cells but needs further investigation. Subsequent delivery of CR108 adjuvants could “inflame” the TME, leading to cDC1 activation (CD40 signaling) and presentation of TAAs to T cells, local chemokine (CCL21 and CXCL13) production, and T and B cell recruitment, as well as TLS formation. The local antitumor immune response triggered by cisplatin+CR108 combo therapy is also corroborated systemically, as evidenced by elevated serum concentration of IFN-Is and TNFα, activation of T cells in dLNs, downregulated Tregs, which may be an important factor in controlling metastasis. It offers a promising avenue for developing more effective cancer immunotherapies, not just for controlling primary tumors but potentially also for preventing the spread of cancer, ultimately improving patient outcomes.

## Methods

### Ethics Approval

All animal experiments were approved by the Committee of Experimental Animals of SHMC (reference number: 202012037S), and carried out in compliance with the ARRIVE guidelines. All mice were sacrificed under euthanasia with isoflurane inhalation at the end of experiments.

### Cell lines and animals

All cell lines, including 4T1 and B16F10, were purchased from Cell Bank/Stem Cell Bank, Chinese Academy of Sciences, and cultivated according to the suppliers’ recommended culture conditions. Female BALB/c mice and C57BL/6 mice (aged 6–8 weeks) were purchased from Beijing Vital River Laboratory Animal Technology Co., Ltd. All animals were fed with a standard rodent diet and provided with water ad libitum. Experimental mice were sourced from the in-house breeding facility, age-matched, and randomly allocated in groups of five to six animals per each cage.

### Tumor implantation, treatment, and survival

3 × 10^5^ B16F10 or 5 × 10^5^ 4T1 tumor cells were injected into the right flank of a C57BL/6 or BALB/c mouse in a unilateral 4T1 or B16F10 tumor model. B16F10 and 4T1 tumors were allowed to grow for ~5 days (to a palpable size of ~4–5 mm in diameter) before treatment with cisplatin (Meilunbio, Shanghai, China) followed by three doses of CR108 (formulated by Advaccine Biopharmaceutics, Suzhou, China) in 7-day intervals, whereas additional tumor cells were implanted into the contralateral side of mice and treated as above in the bilateral model. For the combined therapy with αPD-1 (BioXcell, Cat#BE0273, NH), cisplatin was administered first, followed by 200 μg αPD-1 over five doses, each given at 4-day intervals. The development of tumors was assessed using a digital caliper every two to three days. The dimensions of each tumor were calculated as follows: 0.5 × tumor length × tumor width^2^ (mm^3^). Diameters exceeding 20 mm were deemed indicative of ethical demise.

### Preparation of lymphocytes and TILs

To obtain single-cell suspensions of lymphocytes isolated from lymph nodes, the organs were homogenized with a pestle. Tumors weighing more than 10 mg were collected for TIL analysis. The small pieces of tumor tissue were then incubated for 45 min with collagenase type IV (Sigma, MO, 80 μg/ml) and DNase I (Sigma, 50 U/ml). The tumor pieces were subsequently mechanically dispersed and filtered through a 40-mm sieve, followed by staining and flow cytometry analysis. Cell count/mg = lymphocyte cell counts/tumor weight.

For detection of IFNγ and TNFα production by T cells ex vivo, we followed methods^41^ in which 2×10^6^ lymphocytes from dLN were cultured for 4 h with PMA (50 ng/ml, P8139, Sigma) and ionomycin (500 ng/ml, I0634, Sigma) in the presence of Golgi-stop (1:1000, Cat# 554724, BD Biosciences, CA), followed by staining and flow cytometry analysis.

### Flow cytometry

Single-cell suspensions were immunostained with a panel of fluorochrome-tagged monoclonal antibodies (Supplemental Table 1). For staining cell surface markers, the cells were washed with PBS and then stained for 15 min at room temperature. Following that, the cells were fixed and permeabilized using a Foxp3/transcription factor staining buffer set for the assessment of intracellular antigens (eBiosciences). Intracellular markers were then stained for 1 h at room temperature. In some experiments, precision count beads (Biolegend) were added before acquisition to calculate the absolute cell number. All stained samples were detected on LSRFortessa (BD Biosciences) and analyzed with FlowJo (v10, BD Biosciences).

### Cytokine detection by ELISA

Serum samples were collected at the indicated time to detect cytokines released into the peripheral blood. Peripheral blood samples were centrifuged to separate serum, cytokine concentration in these samples was detected by a pre-coated ELISA kit (Multi Sciences, Hangzhou, China, or Reddot Biotech, Kelowna, Canada) according to the manufacturer’s protocols.

### Histology and microscopy

Tumor tissues were embedded in paraffin after being fixed in 4% paraformaldehyde (PFA)/PBS for more than 24 h. Slice the modified tissue wax block to the thickness of a 4-μm paraffin slice. For the quantification of TLSs, consecutive sections were stained with the H&E staining kit (Cat# 1005, Service Bio). Primary antibodies were employed and detected using corresponding secondary fluorochrome-tagged antibodies (online Supplementary Table 1). Immunofluorescence (IF) staining utilized iF647-Tyramide (G1232, Service Bio) for HRP stainings, followed by a 10-min incubation with TSA in darkness. Microwave-based tissue repair using a citrate solution enabled multiple fluorescent stainings. Nuclei were stained with DAPI (G1012, Service Bio). Sections were scanned using a panoramic scanner (3D HITECH). Alexa Fluor 647, with an excitation range of 608–648 nm and emitting red light within 672–712 nm, was designated as a light pink or yellow shade to distinguish it from Cy3. Brightfield images were developed using DAB (G1212, Service Bio). Additionally, sections were counterstained with hematoxylin and eosin for 5 min. Images were then acquired using a NIKON ECLIPSE C1 fluorescence microscope, scanned with a 3D HITECH panoramic scanner, and analyzed with Caseviewer.

### Single-cell dissociation and processing

Three samples of tumor tissue treated with PBS were labeled as Control Tumor1, Control Tumor2, and Control Tumor3, and one sample treated with cisplatin+CR108 was labeled as cisplatin+CR108-Tumor1. These samples were collected at 25 DPI. Two samples (labeled as cisplatin+CR108-Tumor2 and cisplatin+CR108-Tumor3) from the cisplatin+CR108 combo-treated group were mixed with ten samples from cisplatin+CR108 combo-treated mice to make one sample because it was too small for single-cell sequencing. Three TLS samples were separated surgically and collected from cisplatin+CR108 combo-treated mice on 25 DPI. The tumor tissue of the mouse was stored in MACS^®^ Tissue Storage Solution (PN:130-100-008, Miltenyi Biotec, Bergisch Gladbach, Germany) for about 32 h before it was dissociated into individual cells. Dissociation was performed using the Mouse Tumor Dissociation Kit (PN:130-096-730, Miltenyi Biotec) and gentleMACS™ Octo Dissociator with Heaters (PN:130-096-427, Miltenyi Biotec). Single-cell pellets were washed twice with 1x PBS, centrifuged, and finally resuspended in 1x PBS with 0.04% BSA at a concentration of 700–1200 cells/μL.

### scRNA-seq library preparation and sequencing

The scRNA-seq library was prepared with Chromium Next GEM Single Cell 3’ Reagent Kits v3.1 from 10X Genomics (PN:1000269, 10x Genomics, Inc.) according to the manufacturer’s instructions. About 10,000 cells were loaded on the channel of Single Cell G Chip (PN:1000127, 10x Genomics, Inc.) using Chromium Next GEM Single Cell 3ʹ GEM Kit v3.1 (PN:1000130, 10x Genomics, Inc.) and Chromium Controller (PN:120270, 10x Genomics, Inc.). Single-cell suspensions were mixed with barcoded Single Cell 3ʹ v3.1 Gel Beads and Partitioning Oil to generate nanoliter-scale Gel beads in EMulsion (GEMs). GEMs underwent reverse transcription PCR in a 100 μl volume to obtain full-length cDNA with barcodes using a Thermal Cycler (Long Gene A300). Subsequently, first-strand cDNA was purified using Dynabeads MyOne SILANE (10x Genomics, Inc.) and amplified. The amplified cDNA was utilized to construct a 3ʹ gene expression dual index library using 10x Genomics’ Library Construction Kit (PN:1000196) and Dual Index Kit TT Set A (PN:1000215). The resultant single-cell libraries were sequenced using an Illumina NovaSeq 6000 sequencer.

### scRNA-seq data processing, quality control, and integration

We processed scRNA-seq FASTQ data using the kallisto-bustools workflow with the GRCm39 mouse genome reference. Employing the “lamanno” mode, both spliced and unspliced UMI count matrices were generated. Quality control focused on the spliced UMI count matrix, filtering cells based on empty drops, low mitochondrial expression, and predicted doublets. The retained cells underwent ten sequential steps using Scanpy. These steps included normalization, computation of log1p for the expression count of each gene, employing the top 3000 highly variable genes for principal component analysis (PCA), regression of unwanted variations, data scaling, calculation of the top 50 principal components, sample-level batch effect removal using Harmony, construction of a neighborhood graph with UMAP embedding, and cell clustering via the Leiden algorithm for annotation.

### Major cell type and minor cell statement annotation

We adopted four strategies to annotate major cell types. First, we identified six major cell types based on the expression pattern of typical marker genes, including *Epcam* for epithelial cells, *Plvap* for endothelial cells, *Dcn* for fibroblasts, *Aif1* for the monocyte/macrophage lineage, *Cd3d* for T cells, and *Ms4a1* for B cells. In this process, if a cluster simultaneously has high-expressed marker genes from different kinds of major cell types, this cluster would be filtered out for its potential to be doublets. Second, we performed SingleR to annotate major cell types based on expression correlation between cells and bulk samples collected in the Human Primary Cell Atlas dataset. Third, we used ScType to annotate major cell types based on the expression patterns of marker genes related to various kinds of cell types in the tissue mammary gland collected in the database Cell Taxonomy. Fourth, we used Cell_BLAST to annotate major cell types based on a neural network-based generative model with the reference dataset ALIGNED_Mus_musculus_Mammary_Gland collected in the Cell BLAST database. Last, we harmonized all cell annotations mentioned above and further annotated cell cluster 15 as a plasma cell.

For T cells, we further annotated minor cell statements using ScType with two sources of marker genes. One source was referred to in the database Tumor Immune Single-cell Hub (TISCH). In this step, we also annotated some sub-clusters of T cells as NK cells. The marker genes of naive, inhibitory, cytokines, and costimulatory T cells, and Tregs were selected exactly as previously described^42^. Within the T and NK cell cluster, costimulatory T cells are included 4 costimulatory molecular genes (*Icos*, *Tnfrsf14*, *Tnfrsf9*, and *Cd28*), cytokines T cells, and NK cells are included 11 cytotoxicity associated genes (*Il2*, *Il17a*, *Il17f*, *Lamtor3*, *Gnly*, *Gzmb*, *Prf1*, *Gzmk*, *Ifng*, *Gzma*, *Nkg7*), the inhibitory are five exhausted markers (*Ctla4*, *Havcr2*, *Lag3*, *Pdcd1*, and *Tigit*). Naïve T cells and NK cells include four naïve associated genes (*Ccr7*, *Tcf7*, *Lef1*, and *Sell*), while Treg includes three Treg-associated genes (*Foxp3*, *Il2ra*, and *Ikzfz*).

### RNA velocity analysis

For T cells, we performed RNA velocity analysis using the Python module scVelo based on spliced and unspliced UMI count matrices. Before computing RNA velocity, both matrices were processed in four steps. First, genes with a minimum of 20 spliced and 20 unspliced counts were kept. Second, the total count of each cell was normalized to the median of the count distribution before normalization. Third, the top 2000 highly variable genes were kept. Last, a log1p transformation was conducted for the X matrix of the data object. Two orders of moments, i.e., the mean and uncentered variance of processed spliced and unspliced counts, were then calculated for each cell across its 30 nearest neighbors, where the neighbor graph was built based on Euclidean distances in PCA space. Velocity estimation was next performed with the default stochastic mode, and a velocity graph was constructed based on the cosine similarities between velocities and observed changes in gene expression. Finally, velocity pseudotime was computed as a random walk-based distance from inferred root cells on the velocity graph and visualized as a dot plot in UMAP space. We also performed a density plot for the pseudotime distribution of T cells in different samples or with different cell statements.

### Cell-cell communication analysis

We performed cell communication analysis for different cell statements of T cell, different cell statements of B cell, dendritic cell, and NK cell using CellChat. First, the total expression count of each cell was normalized to 1e4 and log1p transformation was conducted for the expression count of each gene. Then, the probability of cell-cell communication was computed by integrating gene expression count with the interaction between ligand-receptor pairs collected in the database CellChat, using the law of mass action. Next, we visualized the aggregated cell-cell communication network among cell statements and cell types mentioned above with a circle plot. Last, we computed and visualized the relative contribution of each ligand-receptor pair to the signaling pathway related to MHC-I and MHC-II, respectively.

### Analysis of gene expression in Toll-like receptor signaling pathway

Average gene expression levels of single cells belonging to the same groups were calculated using the Seurat (version 4.4.0) “AverageExpression” function. Genes in Toll-like receptor signaling pathway (Mus musculusgene) gene sets of KEGG, which was download from https://www.genome.jp/entry/mmu04620, were used to plot gene average expression and gene set variation analysis (GSVA) enrichment score heatmap by pheatmap package (pheatmap 1.0.12, https://github.com/raivokolde/pheatmap). The GSVA (version 1.50.0) enrichment score was calculated by the GSVA package.

### Statistical analysis

The data were shown as mean ± SEM. The data were analyzed using the unpaired Student’s test between two groups, a one-way ANOVA among multiple groups, and a two-way ANOVA followed by multiple comparisons for multi-factor analysis of variance. A Wilcoxon rank-sum test was performed to compare each cell statement and expression of cytokines (*Ifng*, *Tnf*, and *Il18*) in different treatment groups, and the resulting *P* values were corrected for multiple testing via the method of Benjamini & Hochberg. For animal survival analysis, graphs were generated using the Kaplan–Meier method, and the survival curves were analyzed using log-rank analysis. Statistical analyses were performed on GraphPad Prism 9 (GraphPad Software) except for cell statement ratio comparison. *P* values less than 0.05 were considered significant.

## Supplementary information

Supplementary information

## Data Availability

All data reported in this paper will be shared by contacting the lead contact upon request. Any additional information required to reanalyze the data reported in this work is available from the lead contact upon request. The datasets generated and analyzed during the current study are available in the following repositories and formats: Raw sequencing data: The raw RNA-seq data have been deposited in the NCBI Sequence Read Archive (SRA) under accession numbers: PRJNA1148306, PRJNA1148307, PRJNA1148308, PRJNA1190691, PRJNA1190692, PRJNA1190693, PRJNA1190694, PRJNA1190695, PRJNA1191196.

## References

[1] Gote, V., Nookala, A. R., Bolla, P.K. & Pal, D. Drug resistance in metastatic breast cancer: tumor targeted nanomedicine to the rescue. *Int. J. Mol. Sci.* **22**, 4673 (2021). 10.3390/ijms22094673 PMC8125767 33925129

[2] Bonaventura, P. et al. Identification of shared tumor epitopes from endogenous retroviruses inducing high-avidity cytotoxic T cells for cancer immunotherapy. *Sci. Adv.* **8**, eabj3671 (2022). 35080970 10.1126/sciadv.abj3671 PMC8791462

[3] Rahim, M. K. et al. Dynamic CD8(+) T cell responses to cancer immunotherapy in human regional lymph nodes are disrupted in metastatic lymph nodes. *Cell* **186**, 1127–1143 e1118 (2023). 36931243 10.1016/j.cell.2023.02.021 PMC10348701

[4] Haanen, J. Converting cold into hot tumors by combining immunotherapies. *Cell* **170**, 1055–1056 (2017). 28886376 10.1016/j.cell.2017.08.031

[5] Sharma, P., Hu-Lieskovan, S., Wargo, J. A. & Ribas, A. Primary, adaptive, and acquired resistance to cancer immunotherapy. *Cell* **168**, 707–723 (2017). 28187290 10.1016/j.cell.2017.01.017 PMC5391692

[6] Sterner, R. C. & Sterner, R. M. CAR-T cell therapy: current limitations and potential strategies. *Blood Cancer J.* **11**, 69 (2021). 33824268 10.1038/s41408-021-00459-7 PMC8024391

[7] Lybaert, L. et al. Challenges in neoantigen-directed therapeutics. *Cancer Cell* **41**, 15–40 (2023). 36368320 10.1016/j.ccell.2022.10.013

[8] Chen, J. et al. In situ cancer vaccination using lipidoid nanoparticles. *Sci. Adv.* **7**, eabf1244 (2021). 10.1126/sciadv.abf1244 PMC8099179 33952519

[9] Karime, C. et al. Tilsotolimod: an investigational synthetic toll-like receptor 9 (TLR9) agonist for the treatment of refractory solid tumors and melanoma. *Expert Opin. Investig. Drugs* **31**, 1–13 (2022). 34913781 10.1080/13543784.2022.2019706

[10] Anwar, M. A., Shah, M., Kim, J. & Choi, S. Recent clinical trends in Toll-like receptor targeting therapeutics. *Med Res Rev.* **39**, 1053–1090 (2019). 30450666 10.1002/med.21553 PMC6587958

[11] Fucikova, J. et al. Detection of immunogenic cell death and its relevance for cancer therapy. *Cell Death Dis.* **11**, 1013 (2020). 33243969 10.1038/s41419-020-03221-2 PMC7691519

[12] Walshaw, R. C., Honeychurch, J., Choudhury, A. & Illidge, T. M. Toll-like receptor agonists and radiation therapy combinations: an untapped opportunity to induce anticancer immunity and improve tumor control. *Int J. Radiat. Oncol. Biol. Phys.* **108**, 27–37 (2020). 32339645 10.1016/j.ijrobp.2020.04.020

[13] Bahmani, B. et al. Intratumoral immunotherapy using platelet-cloaked nanoparticles enhances antitumor immunity in solid tumors. *Nat. Commun.* **12**, 1999 (2021). 33790276 10.1038/s41467-021-22311-z PMC8012593

[14] Grabosch, S. et al. Cisplatin-induced immune modulation in ovarian cancer mouse models with distinct inflammation profiles. *Oncogene* **38**, 2380–2393 (2019). 30518877 10.1038/s41388-018-0581-9 PMC6440870

[15] de Biasi, A. R., Villena-Vargas, J. & Adusumilli, P. S. Cisplatin-induced antitumor immunomodulation: a review of preclinical and clinical evidence. *Clin. Cancer Res.* **20**, 5384–5391 (2014). 25204552 10.1158/1078-0432.CCR-14-1298 PMC4216745

[16] Sautes-Fridman, C., Petitprez, F., Calderaro, J. & Fridman, W. H. Tertiary lymphoid structures in the era of cancer immunotherapy. *Nat. Rev. Cancer* **19**, 307–325 (2019). 31092904 10.1038/s41568-019-0144-6

[17] Kang, W. et al. Tertiary lymphoid structures in cancer: the double-edged sword role in antitumor immunity and potential therapeutic induction strategies. *Front. Immunol.* **12**, 689270 (2021). 34394083 10.3389/fimmu.2021.689270 PMC8358404

[18] Helmink, B. A. et al. B cells and tertiary lymphoid structures promote immunotherapy response. *Nature* **577**, 549–555 (2020). 31942075 10.1038/s41586-019-1922-8 PMC8762581

[19] Cabrita, R. et al. Tertiary lymphoid structures improve immunotherapy and survival in melanoma. *Nature* **577**, 561–565 (2020). 31942071 10.1038/s41586-019-1914-8

[20] Aoyama, S., Nakagawa, R., Mule, J. J. & Mailloux, A. W. Inducible tertiary lymphoid structures: promise and challenges for translating a new class of immunotherapy. *Front. Immunol.* **12**, 675538 (2021). 34054863 10.3389/fimmu.2021.675538 PMC8160316

[21] Fridman, W. H. et al. B cells and tertiary lymphoid structures as determinants of tumour immune contexture and clinical outcome. *Nat. Rev. Clin. Oncol.* **19**, 441–457 (2022). 35365796 10.1038/s41571-022-00619-z

[22] Chaurio, R. A. et al. TGF-beta-mediated silencing of genomic organizer SATB1 promotes Tfh cell differentiation and formation of intra-tumoral tertiary lymphoid structures. *Immunity* **55**, 115–128 e119 (2022). 35021053 10.1016/j.immuni.2021.12.007 PMC8852221

[23] Rodriguez, A. B. et al. Immune mechanisms orchestrate tertiary lymphoid structures in tumors via cancer-associated fibroblasts. *Cell Rep.* **36**, 109422 (2021). 34289373 10.1016/j.celrep.2021.109422 PMC8362934

[24] Peske, J. D. et al. Effector lymphocyte-induced lymph node-like vasculature enables naive T-cell entry into tumours and enhanced anti-tumour immunity. *Nat. Commun.* **6**, 7114 (2015). 25968334 10.1038/ncomms8114 PMC4435831

[25] Galon, J. & Bruni, D. Approaches to treat immune hot, altered and cold tumours with combination immunotherapies. *Nat. Rev. Drug Discov.* **18**, 197–218 (2019). 30610226 10.1038/s41573-018-0007-y

[26] Giraldo, N. A. et al. Orchestration and prognostic significance of immune checkpoints in the microenvironment of primary and metastatic renal cell cancer. *Clin. Cancer Res.* **21**, 3031–3040 (2015). 25688160 10.1158/1078-0432.CCR-14-2926

[27] Hodi, F. S. et al. Improved survival with ipilimumab in patients with metastatic melanoma. *N. Engl. J. Med.* **363**, 711–723 (2010). 20525992 10.1056/NEJMoa1003466 PMC3549297

[28] Lin, M. J. et al. Cancer vaccines: the next immunotherapy frontier. *Nat. Cancer* **3**, 911–926 (2022). 35999309 10.1038/s43018-022-00418-6

[29] Fridman, W. H. et al. Tertiary lymphoid structures and B cells: an intratumoral immunity cycle. *Immunity* **56**, 2254–2269 (2023). 37699391 10.1016/j.immuni.2023.08.009

[30] Cai, D. et al. Turning tertiary lymphoid structures (TLS) into hot spots: values of TLS in gastrointestinal tumors. *Cancers* **15**, 367 (2023). 10.3390/cancers15020367 PMC9856964 36672316

[31] Zhu, S., Pabla, N., Tang, C., He, L. & Dong, Z. DNA damage response in cisplatin-induced nephrotoxicity. *Arch. Toxicol.* **89**, 2197–2205 (2015). 26564230 10.1007/s00204-015-1633-3 PMC4734632

[32] Cohen, S. M. et al. Subcutaneous delivery of nanoconjugated doxorubicin and cisplatin for locally advanced breast cancer demonstrates improved efficacy and decreased toxicity at lower doses than standard systemic combination therapy in vivo. *Am. J. Surg.* **202**, 646–652 (2011). 21982998 10.1016/j.amjsurg.2011.06.027 PMC5198781

[33] Zhang, Y. et al. FimH confers mannose-targeting ability to Bacillus Calmette-Guerin for improved immunotherapy in bladder cancer. *J. Immunother. Cancer* **10**, e003939 (2022). 10.1136/jitc-2021-003939 PMC8971803 35361729

[34] Vacchelli, E. et al. Trial watch: FDA-approved Toll-like receptor agonists for cancer therapy. *Oncoimmunology* **1**, 894–907 (2012). 23162757 10.4161/onci.20931 PMC3489745

[35] Sagiv-Barfi, I. et al. Eradication of spontaneous malignancy by local immunotherapy. *Sci. Transl. Med.* **10**, eaan4488 (2018). 10.1126/scitranslmed.aan4488 PMC5997264 29386357

[36] Zawit, M. et al. Current status of intralesional agents in treatment of malignant melanoma. *Ann. Transl. Med.* **9**, 1038 (2021). 34277838 10.21037/atm-21-491 PMC8267328

[37] He, Y. et al. STING protein-based in situ vaccine synergizes CD4(+) T, CD8(+) T, and NK cells for tumor eradication. *Adv. Health. Mater.* **12**, e2300688 (2023). 10.1002/adhm.202300688 PMC10964211 37015729

[38] Zhao, J. et al. In situ activation of STING pathway with polymeric SN38 for cancer chemoimmunotherapy. *Biomaterials* **268**, 120542 (2021). 33249316 10.1016/j.biomaterials.2020.120542

[39] Gu-Trantien, C. et al. CXCL13-producing TFH cells link immune suppression and adaptive memory in human breast cancer. *JCI Insight* **2**, e91487 (2017). 10.1172/jci.insight.91487 PMC5453706 28570278

[40] Gu-Trantien, C. et al. CD4(+) follicular helper T cell infiltration predicts breast cancer survival. *J. Clin. Invest.* **123**, 2873–2892 (2013). 23778140 10.1172/JCI67428 PMC3696556

[41] Zhang, M. et al. CCL7 recruits cDC1 to promote antitumor immunity and facilitate checkpoint immunotherapy to non-small cell lung cancer. *Nat. Commun.* **11**, 6119 (2020). 33257678 10.1038/s41467-020-19973-6 PMC7704643

[42] Sun, Y. et al. Single-cell landscape of the ecosystem in early-relapse hepatocellular carcinoma. *Cell* **184**, 404–421.e416 (2021). 33357445 10.1016/j.cell.2020.11.041

