# Supplementary material for: TLR7/8/9 agonists and low-dose cisplatin synergistically promotes tertiary lymphatic structure formation and antitumor immunity
Source: NPJ Vaccines. 2025 Jan 19;10:13. doi: 10.1038/s41541-024-01055-z (PMC11742977; doi:10.1038/s41541-024-01055-z)
Supplement: Supplementary file 1 — Supplementary information [file 41541_2024_1055_MOESM1_ESM.docx]

Supplementary Materials for

**In situ vaccination with TLR7/8/9 agonist and low-dose cisplatin synergistically promotes tertiary lymphatic structures formation and antitumor immunity**

Shuting Wu *et al.*

*Corresponding author. Email: [bwang3@fudan.edu.cn](file:///E:\Users\binwang\Library\Containers\com.tencent.WeWorkMac\Data\Documents\Profiles\AF5A8D35A95E52EEBEC58DBD5AC07CCA\Caches\Files\2024-03\9b296908215b503a436449b61d6327fb\bwang3@fudan.edu.cn) (Bin Wang); [gaoxiaoming@advaccine.com](mailto:gaoxiaoming@advaccine.com) (Xiaoming Gao)

**This file includes:**

Supplementary Tables

Table 1 Monoclonal antibodies used for flow cytometry and histology.

Table 2 Cell type annotation and cluster identification.

Supplementary Figures

Figure 1. The anti-tumor efficacy depends on the optimal dosage of cisplatin and CR108 in the 4T-1 model.

Figure 2. Analysis of tumor samples by Hematoxylin-eosin (HE) staining.

Figure 3. Immunohistochemical and immunofluorescence analysis of TLS in cisplatin+CR108 combo-treated mice.

Figure 4 Cell statement and subtype distribution and RNA velocity analysis of T and NK cells.

Figure 5 Characterizing cell-cell interactions within TLS.

Figure 6. Quantification analysis of TILs.

Figure 7. Effects of T cells mediated response in cisplatin+CR108 combo therapy on antitumor activity.

Figure 8. Functional analysis of TILs.

Figure 9. Assessment of TLR signal pathway in DCs and T cells after cisplatin+CR108 combo therapy.

**Supplementary Tables**

**Supplementary Table 1. Monoclonal antibodies were used for flow cytometry, histology and cells depletion.**

| **REAGENT** | **SOURCE** | **IDENTIFIER** |
| --- | --- | --- |
| **Antibodies** |  |  |

| Anti-CD3 Rabbit pAb | Servicebio | Cat# GB111337 |
| --- | --- | --- |
| Anti-CD11c Rabbit pAb | Servicebio | Cat# GB11059 |
| Recombinant Anti-CD4 antibody | Servicebio | Cat# GB15064 |
| Recombinant Anti-CD8α Rabbit antibody | Servicebio | Cat# GB15068 |
| Anti-Foxp3 Rabbit pAb | Servicebio | Cat# GB112325 |
| Biotin anti-mouse/human PNAd Antibody (MECA-79) | Biolegend | Cat# 120803 |
| CXCR5 Recombinant Rabbit Monoclonal Antibody (JB11-40) | eBiosciences | Cat# JB11-40 |
| CXCL13/BLC/BCA-1 Antibody | Novus Biologicals | Cat# AF470 |
| CD45R (B220) Monoclonal Antibody (RA3-6B2) | eBiosciences | Cat# 14-0452-81 |
| CCL21/6Ckine Antibody | Novus Biologicals | Cat# AF457-SP |
| CCL19/MIP-3 beta Antibody | Novus Biologicals | Cat# AF880-SP |
| Purified anti-mouse Podoplanin Antibody (8.1.1) | Biolegend | Cat# 127401 |
| cyanin 3 Goat anti-rat IgG (Cy3) | Servicebio | Cat# GB21302 |
| cyanin 3 Goat anti-rabbit IgG (Cy3) | Servicebio | Cat# GB21303 |
| HRP Goat anti-mouse IgG | Servicebio | Cat# GB23301 |
| HRP Rabbit anti-goat IgG | Servicebio | Cat# GB23204 |
| HRP Goat anti-rabbit IgG | Servicebio | Cat# GB23303 |
| HRP Goat anti-rat IgG | Servicebio | Cat# GB23302 |
| 488 Goat anti-rabbit IgG | Servicebio | Cat# GB25303 |
| Alexa Flour 488 Goat anti-Rat IgM | eBiosciences | Cat# A-21212 |
| Alexa Fluor 647 Goat anti-Syrian hamster IgG H&L | abcam | Cat# ab180117 |
| Percp cy5.5 anti-mouse CD45(30-F11) | BioLegend | Cat# 103131 |
| Brilliant Violet 510™ anti-mouse CD4 Antibody(GK1.5) | BioLegend | Cat# 100449 |
| PE anti-mouse CD8a Antibody (53-6.7) | BioLegend | Cat# 100708 |
| Percp cy5.5 anti-mouse CD8a Antibody (53-6.7) | BioLegend | Cat# 100734 |
| APC anti-mouse CD8a Antibody (53-6.7) | BioLegend | Cat# 100712 |
| PE anti-mouse CD40 Antibody (3/23) | BioLegend | Cat# 124610 |
| [Brilliant Violet 510™ anti-mouse/human CD11b Antibody](https://www.biolegend.com/en-us/products/brilliant-violet-510-anti-mouse-human-cd11b-antibody-7993) (M1/70) | BioLegend | Cat# 101263 |
| Brilliant Violet 421™ anti-mouse CD11c Antibody | BioLegend | Cat# 117343 |
| [APC anti-mouse CD279 (PD-1) Antibody](https://www.biolegend.com/en-us/products/apc-anti-mouse-cd279-pd-1-antibody-6672) (RMP1-30) | BioLegend | Cat# 109112 |
| PerCP/Cyanine5.5 anti-mouse CD185 (CXCR5) Antibody (L138D7) | BioLegend | Cat# 145508 |
| [Brilliant Violet 421™ anti-mouse CD223 (LAG-3) Antibody](https://www.biolegend.com/en-us/products/brilliant-violet-421-anti-mouse-cd223-lag-3-antibody-13008) (C9B7W) | BioLegend | Cat# 125221 |
| [PE/Cyanine7 anti-mouse CD366 (Tim-3) Antibody](https://www.biolegend.com/en-us/products/pe-cyanine7-anti-mouse-cd366-tim-3-antibody-13929) (B8.2C12) | BioLegend | Cat# 134010 |
| CD3e Monoclonal Antibody (145-2C11), FITC | eBiosciences | Cat# 14-0031-86 |
| CD45R (B220) Monoclonal Antibody (RA3-6B2), eFluor™ 450 | eBiosciences | Cat# 48-0452-82 |
| TNF alpha Monoclonal Antibody (MP6-XT22), FITC | eBiosciences | Cat# 11-7321-82 |
| Brilliant Violet 421™ anti-mouse IFN-γ Antibody | BioLegend | Cat# 505830 |
| FOXP3 Monoclonal Antibody (FJK-16s), PE | eBiosciences | Cat# 12-5773-82 |
| fixable viability dye eFluor 780 | eBiosciences | Cat# 65-0865-14 |
| Precision Count Beads™ | Biolegend | Cat# 424902 |
| InVivoMAb anti-mouse CD4 (GK1.5) | BioXcell | Cat# BE0003 |
| InVivoMAb anti-mouse CD8α (2.43) | BioXcell | Cat# BE0061 |

**Supplementary Table 2. Cell type annotation and cluster identification.**

| Cell Type Annotation | Cluster Identification |
| --- | --- |
| T cells | 1; 6; 11; 12; 14 |
| Tumor-originating cells | 0; 2; 5; 8; 21 |
| Endothelial cells | 18 |
| Fibroblasts | 10 |
| B cells | 3; 16; 24 |
| Plasma cells | 15 |
| Monocytes and macrophages | 4; 17 |
| Dendritic cells | 19 |
| Granulocytes | 9 |
| Binucleated cells | 7; 20; 13 |
| Cells with high proportion expression of mitochondrial genes | 13 |
| Rare islet cells | 22; 23; 25; 26 |

Note:

Cluster Identification: The t-Distributed Stochastic Neighbor Embedding (t-SNE) plot of all cells in the sample, with each color representing a different cell cluster, where the Arabic numerals correspond to the content of Figure S4a.

**Supplementary Figures**

**Supplementary Figure 1.**

**a**

**d e**

**b c**


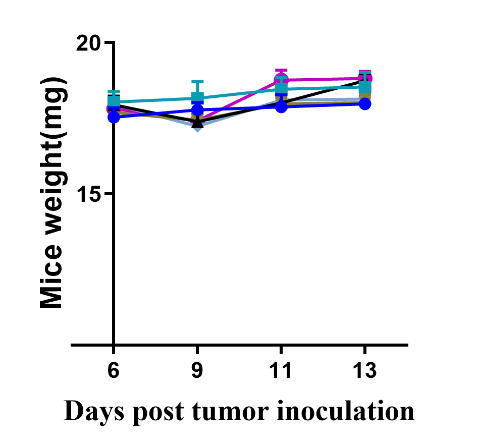


**f g**


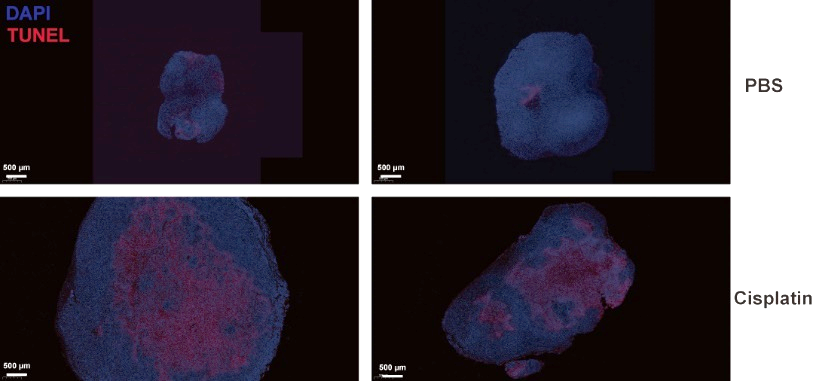


**h**

**Supplementary Figure 1.** **The anti-tumor efficacy depends on the optimal dosage of cisplatin and CR108 in the 4T-1 model.**

(**a**) BALB/c mice (PBS, n=5; others, n=6) were injected s.c. with 5 × 10^5^ viable 4T1 cells. Peritumoral cisplatin was administered in the right flanks, when tumors reached a palpable size of approximately 4–5 mm in diameter. Tumor growth and body weight were monitored daily (right panel). (**b-c**) BALB/c mice were injected s.c. with 5 × 10^5^ viable 4T1 cells. In the right flanks and for tumors reaching a palpable size of approximately 4–5 mm in diameter, peritumoral cisplatin was administered, followed by 3 doses of CR108 (7 days interval, as shown in Fig 1a). Mice that received either PBS, cisplatin, or CR108 alone were included as controls. Primary tumor growth curves (**b**) and tumor weight at tumor monitor endpoint (**c**) under 0.5 μg/kg or 2 μg/kg cisplatin with 10 μg/mouse CR108. PBS and 2 μg/kg cisplatin, n=6; others, n=7. (**d-f**) The design of the 4T1 tumor model with dose escalation of CR108 and fixed dose of cisplatin was similar to that of (**b-c**). Primary tumor growth curves (**d**), tumor weight at endpoint of monitoring (**e**), and body weight curves (**f**) were assessed in mice receiving 2 μg/kg cisplatin with 10 μg/mouse or 50 μg/mouse CR108. The number of animals per group was as follows: PBS, n=9; 2 μg/kg cisplatin, 2 μg/kg cisplatin + 10 μg/mouse CR108 and 10 μg/mouse CR108, n = 6; others, n=8. (**g**) The design of 4T1 tumor model with dose escalation of cisplatin and fixed dose of CR108 was was similarly to that of (**b-c**). Primary tumor growth curves were assessed under 2 μg/kg or 2 mg/kg cisplatin with 50 μg/mouse CR108. The number of animals per group was as follows: 2 μg/kg cisplatin + 50 μg CR108, n=6; others, n=5. (**h**) 4T-1 tumors were collected from PBS and 2 μg/kg cisplatin treated group (24 hours after treatment), fixed and sectioned. These sections were stained with TUNEL to reveal apoptosis and DAPI as an indicator for nuclei. Statistics significance was determined using one-way and two-way ANOVA. Error bars represent mean ± SEM. *P < 0.05, **P < 0.01, ***P < 0.001, ****P < 0.0001.

**Supplementary Figure 2.**

**a**


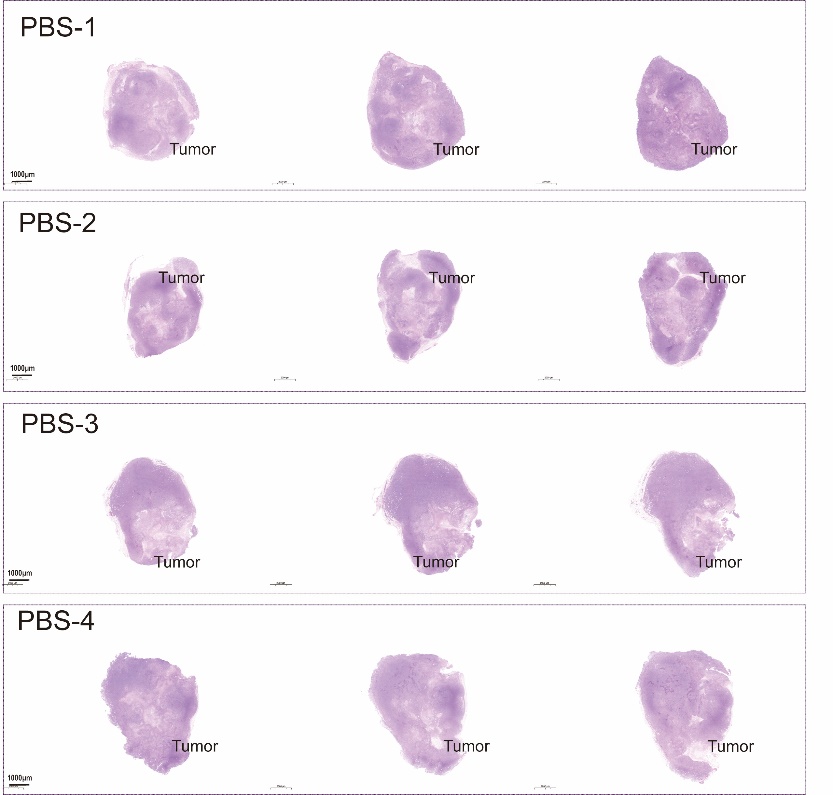

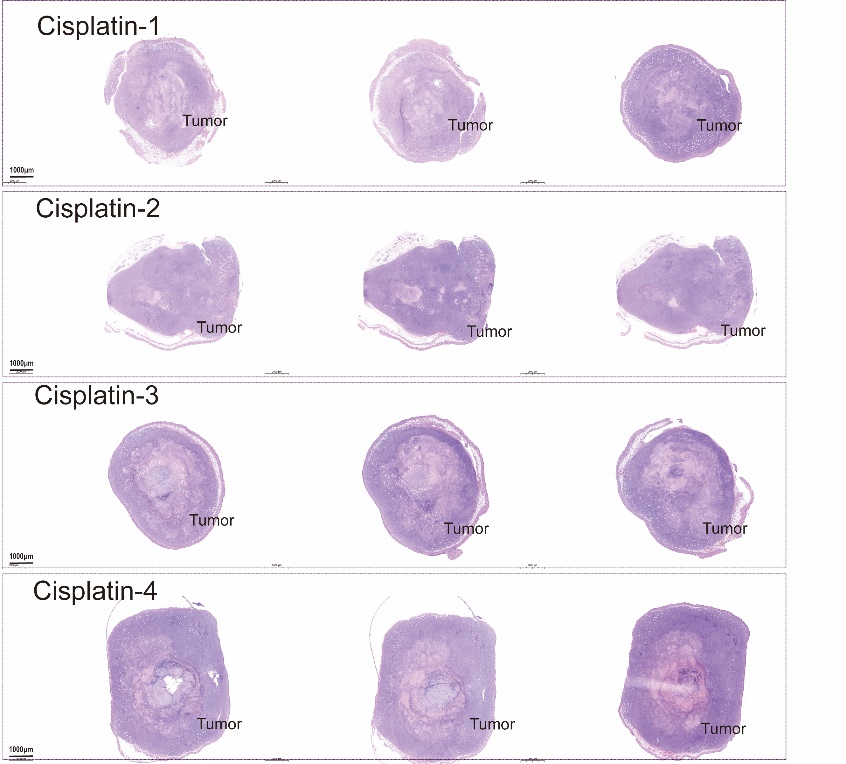


**b**

**c**


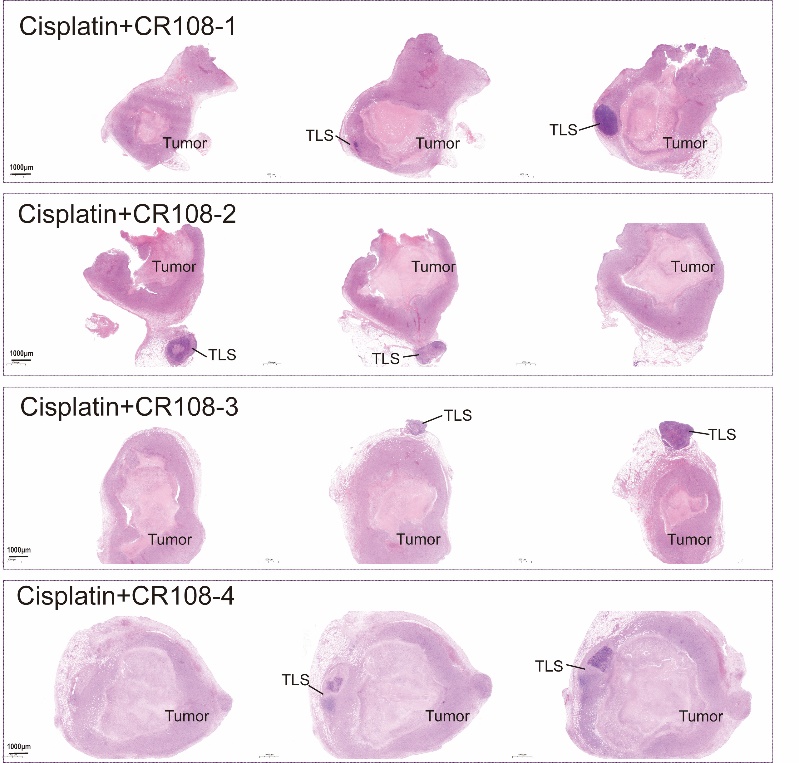


**d**


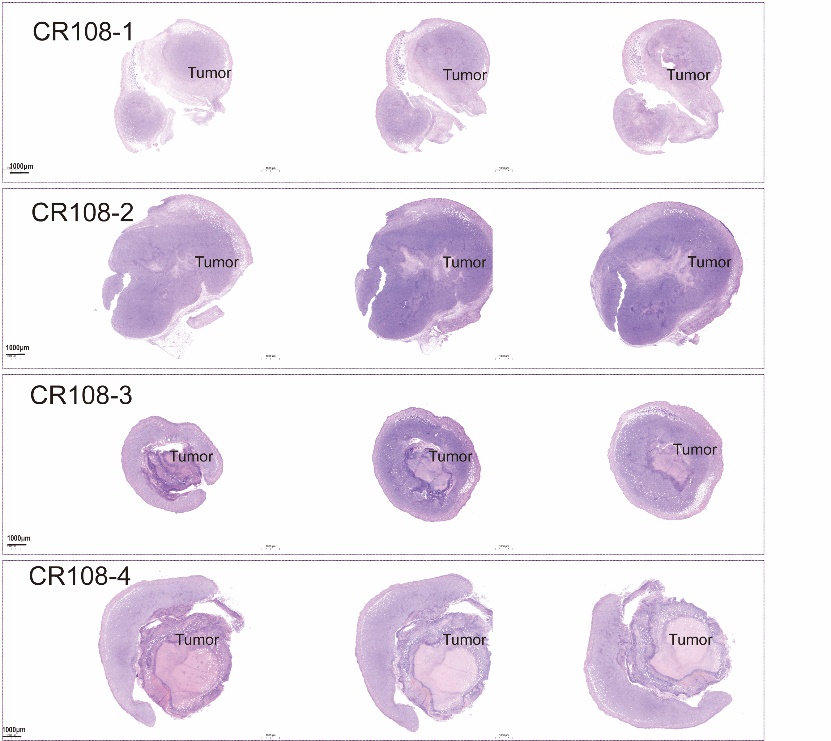


**Supplementary Figure 2. Analysis of tumor samples by Hematoxylin-eosin (HE) staining.**

(**a-d**) Four tumors were collected randomly from tumor bearing animals from each treatment group, fixed, and three sections (1 mm apart) of each tumor block were obtained. These sections were stained with Hematoxylin-eosin to reveal any morphological changes on 28DPI with the 4T1 model. (**a**) PBS treated group, PBS-1 to PBS-4; (**b**) the cisplatin treated group, cisplatin-1 to cisplatin-4; (**c**) the cisplatin+CR108 combo treated group, cisplatin+CR108-1 to Cisplatin+CR108-4; (**d**) CR108 treated group, CR108-1 to CR108-4. TLS were indicated by the arrows.

**Supplementary Figure 3.**

**a**


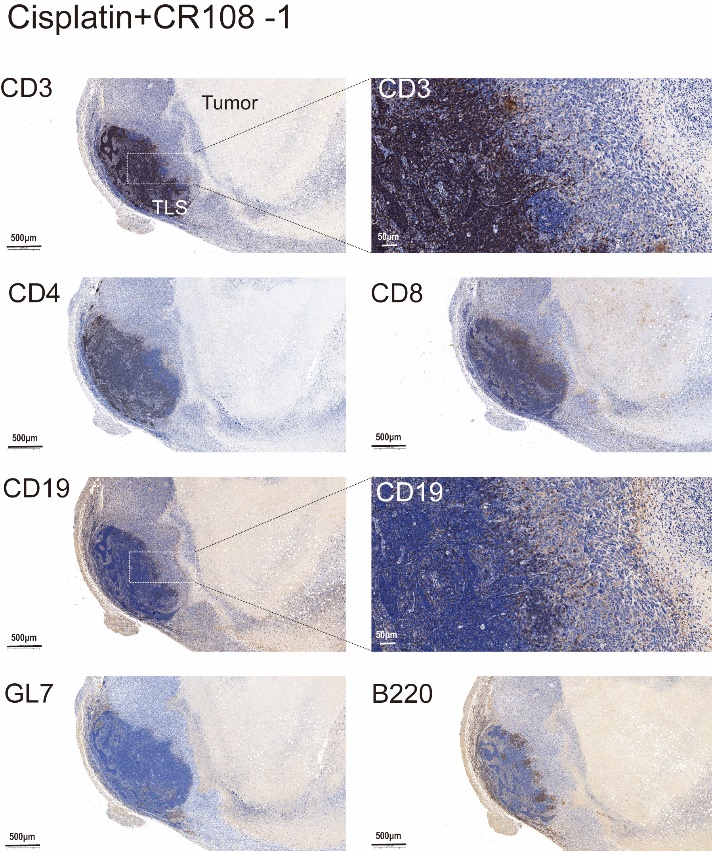


**b**


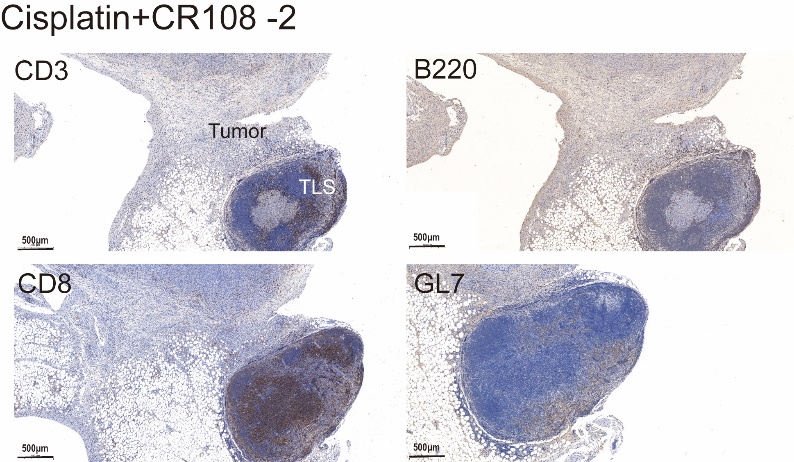


**c**


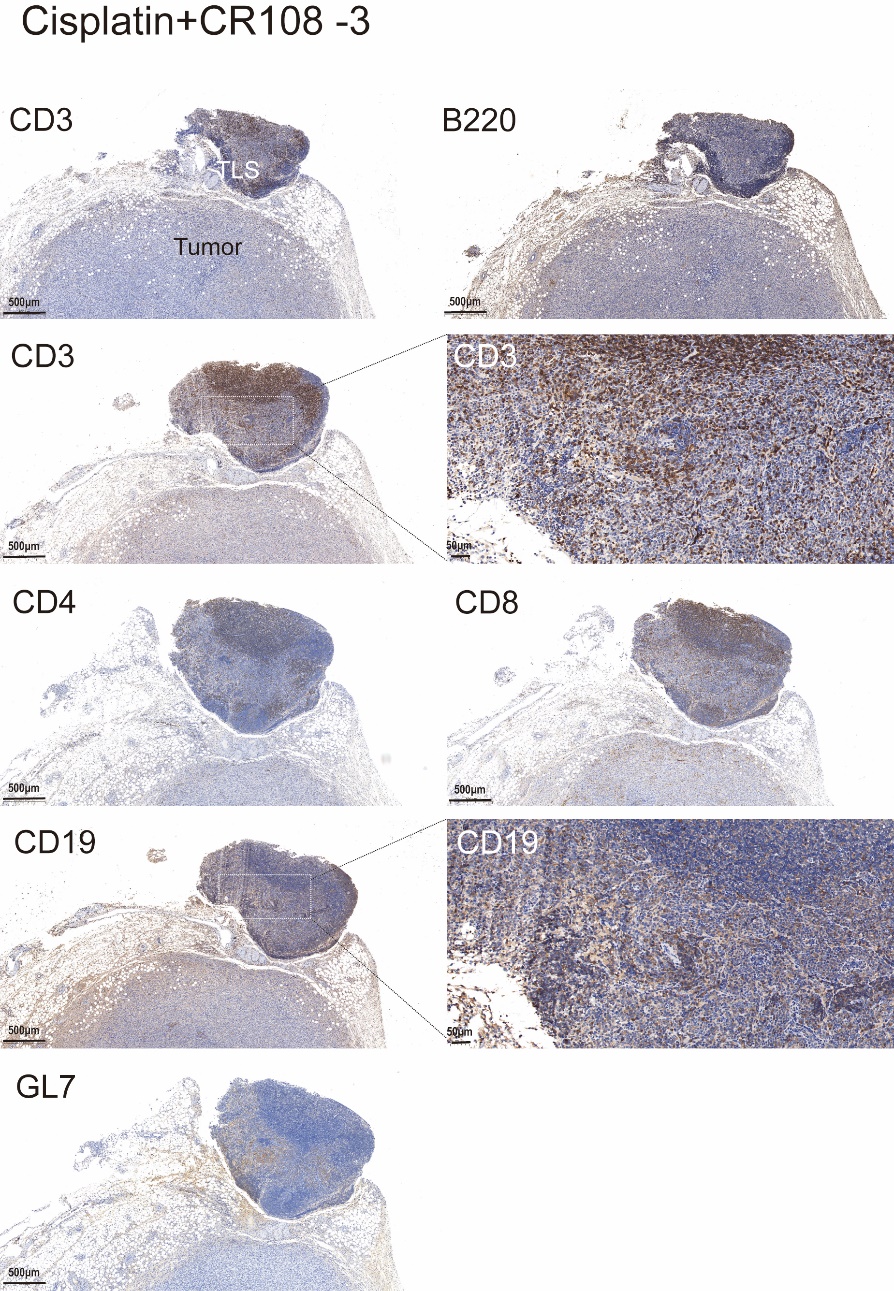


**d**


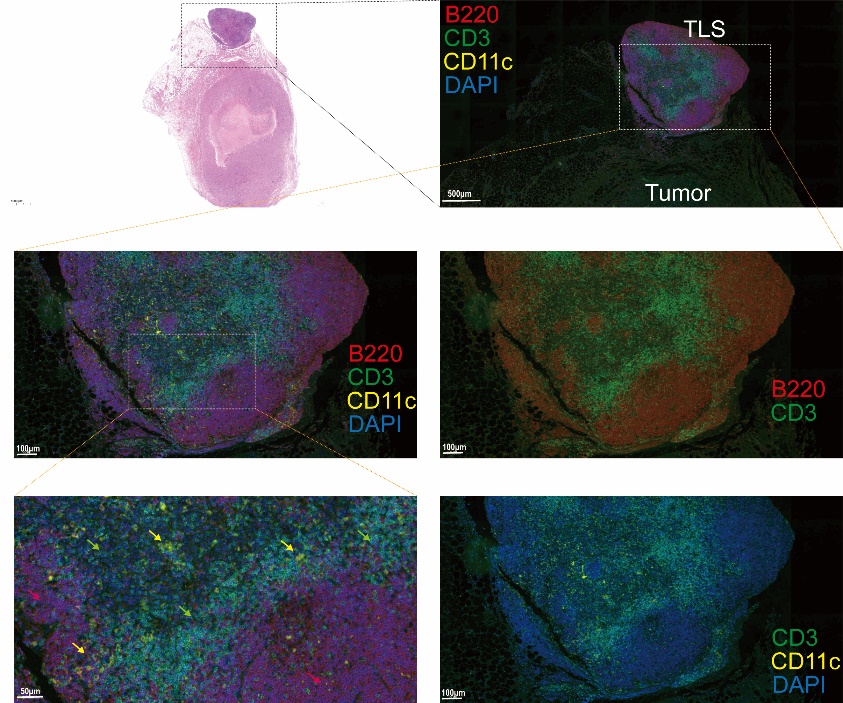


**
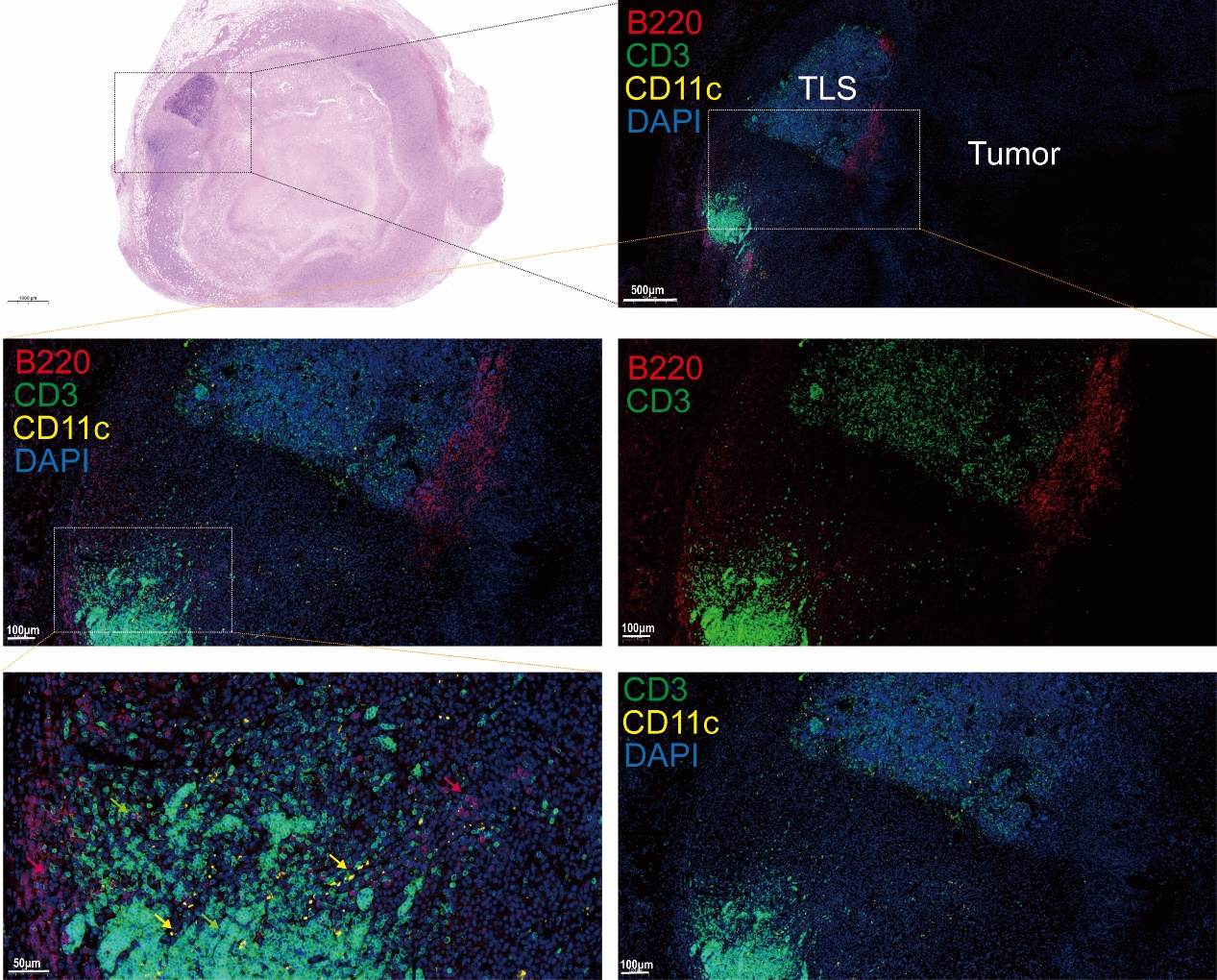
**

**e**

**f**


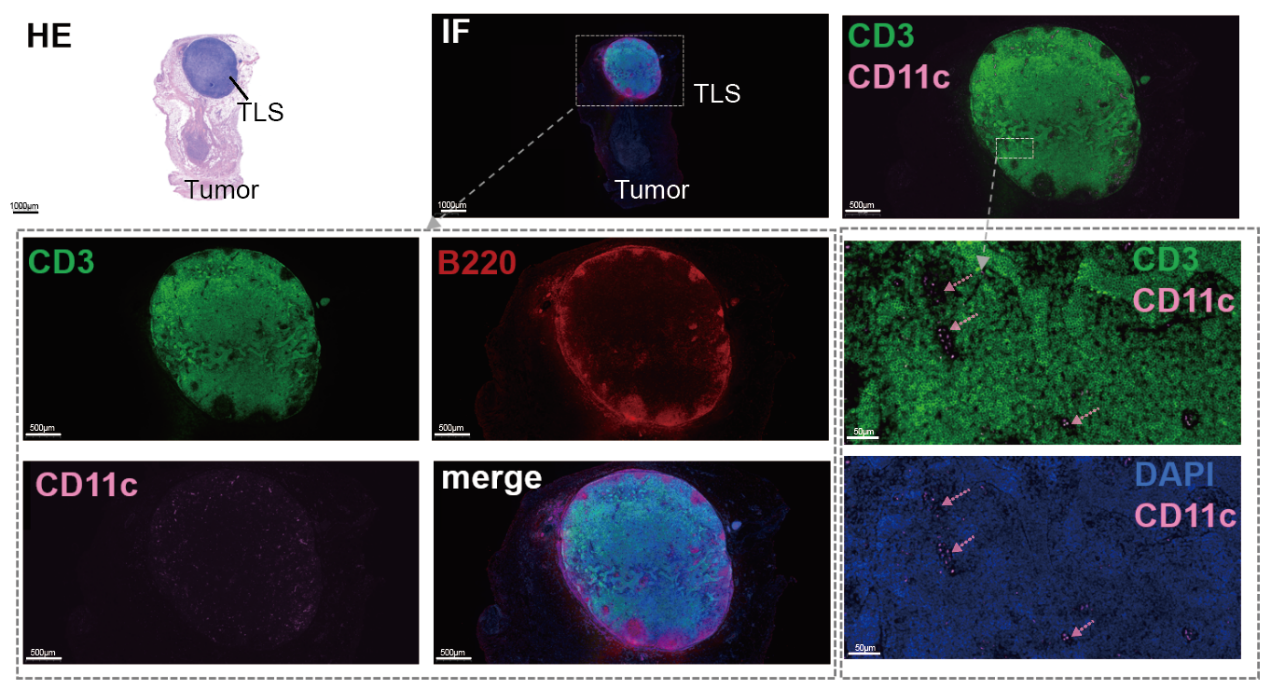


**g**


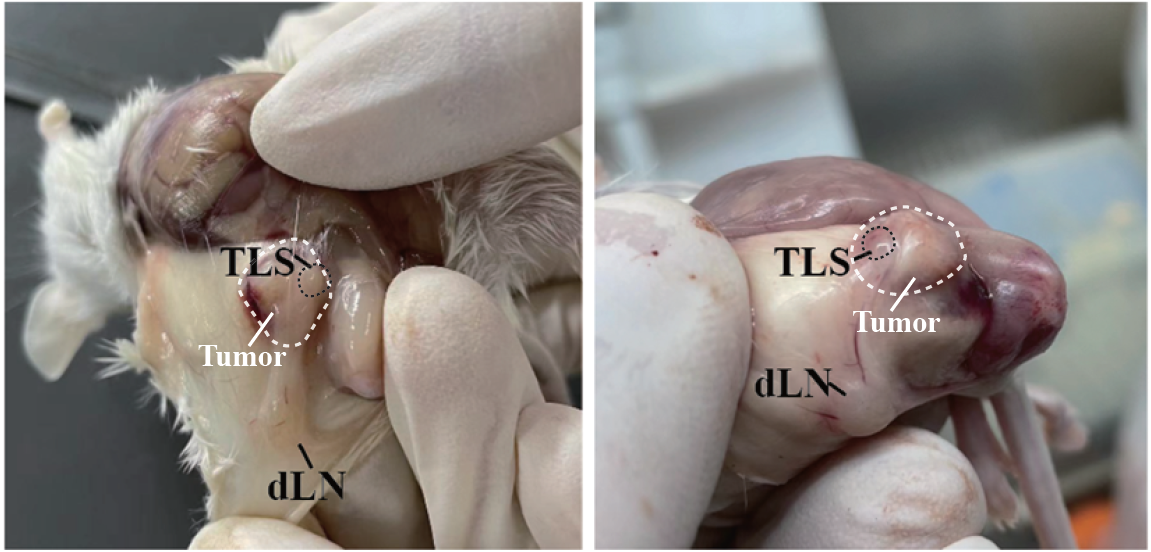


**Supplementary Figure 3. Immunohistochemical and immunofluorescence analysis of TLS in cisplatin+CR108 combo-****treated mice.**

Three randomly collected tumor samples from each cisplatin+CR108 combo-treated mice (cisplatin+CR108-1, -2, and -3) were removed and fixed. A serial sections of each tumor block were used for immunohistochemical (**a-c**) and immunofluorescence (**d-e**) analysis to reveal the structure of the tumor and TLS on 28DPI. Fluorescent channels and corresponding targets were labeled in each image. The green, red, and yellow fluorescence channels in the image of (**d-e**) and green, red and magenta in the image (**f**) represented CD3, B220, and CD11c, respectively. The arrow indicated the location of DCs. Blue represents DAPI staining for nuclei. (**~~g~~**) Gross assessment of TLS and tumor was recorded photographically.

**Supplementary Figure 4.**


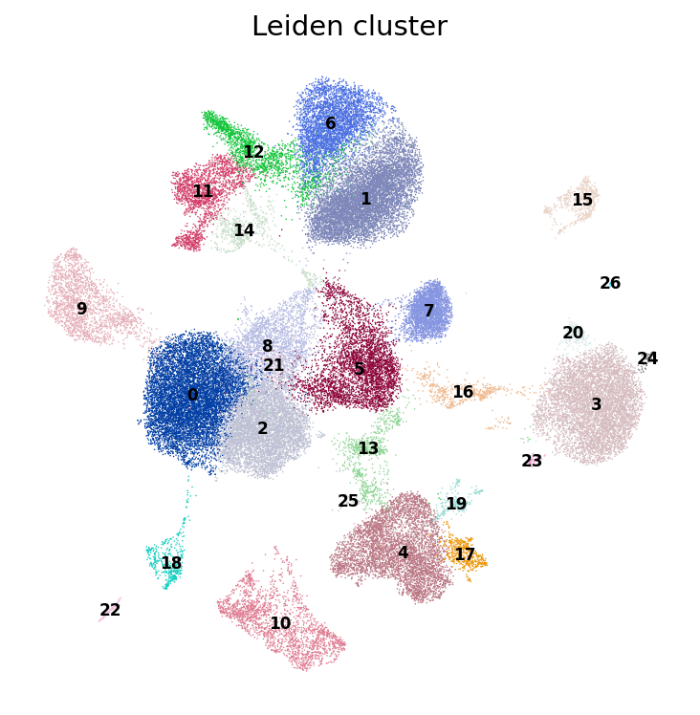


**a**


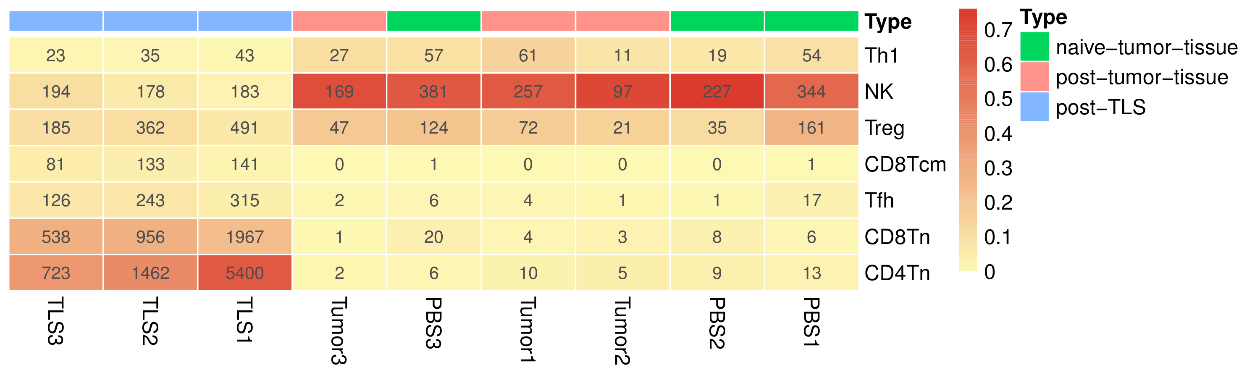


**b**


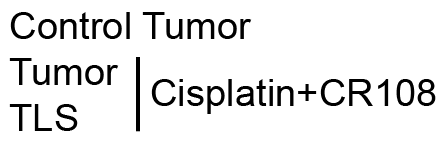


**c**


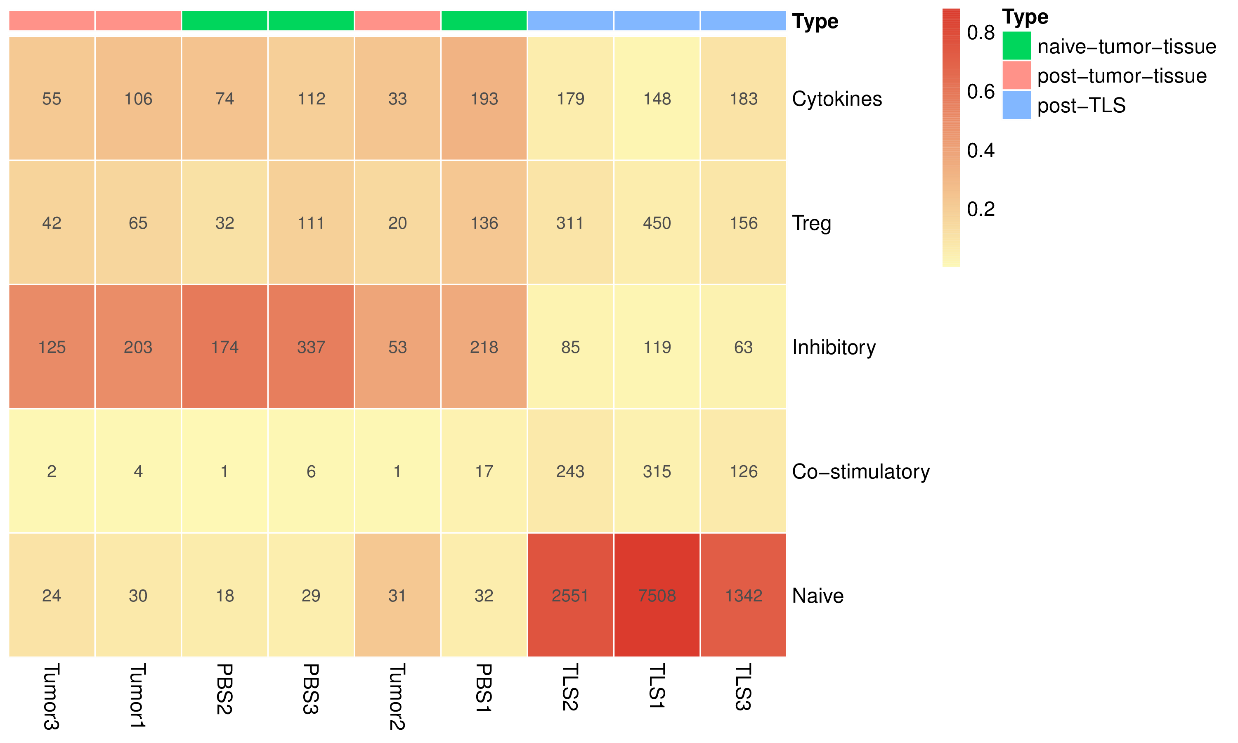

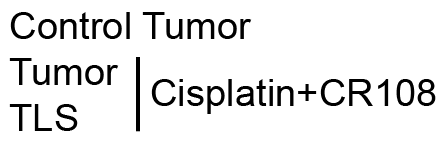

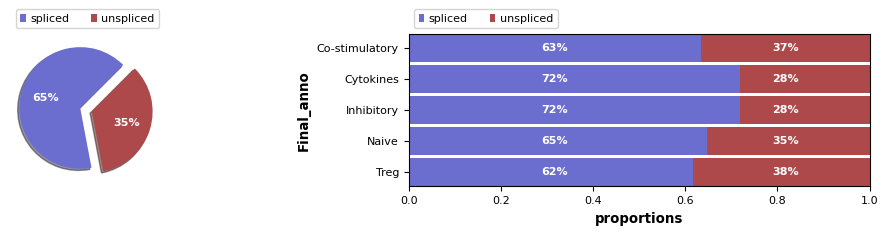

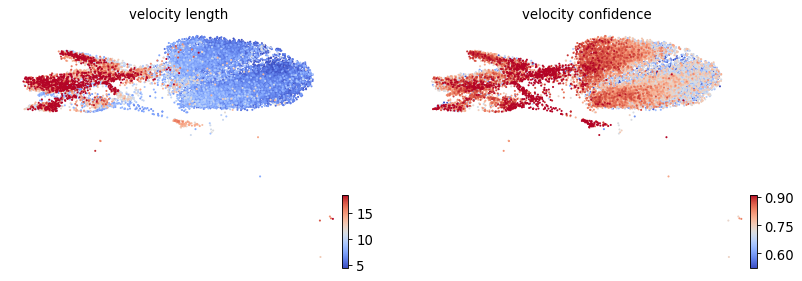


**d**


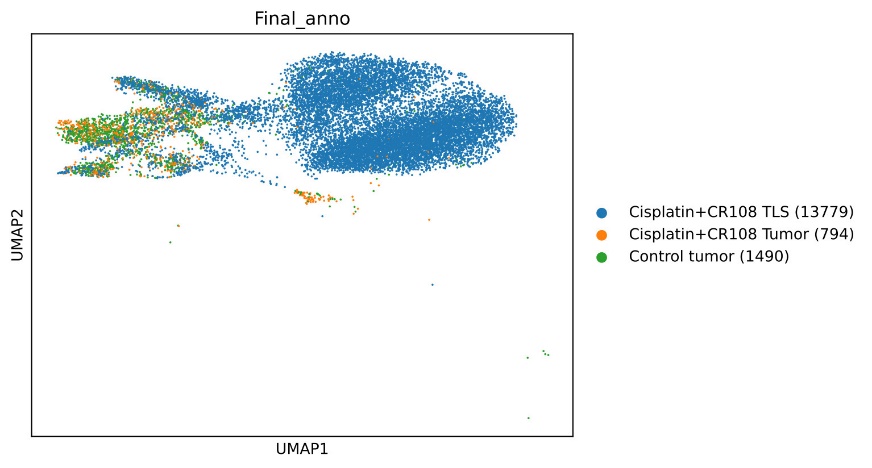


**e**

**Supplementary Figure 4.** **Cell statement and subtype distribution and RNA velocity analysis of T and NK cells.**

**(a)** The t-Distributed Stochastic Neighbor Embedding (t-SNE) plot of all cells in the sample, where each color represents a different cell cluster and Arabic numerals correspond to the content of Table S2. **(b)** Subtype distribution of T and NK cells in tumor samples from PBS-treated and cisplatin+CR108 combo-treated 4T-1 mice and TLS samples from cisplatin+CR108 combo-treated 4T-1 mice. (**c**) Cell expression distribution of T and NK cells in tumor samples from PBS-treated and cisplatin+CR108 combo-treated 4T-1 mice and TLS samples from cisplatin+CR108 combo-treated 4T-1 mice. (**d**) RNA velocity analysis of T and NK cells. The top panel showed the ratio between spliced and unspliced RNAs in the total number of T and NK cells and those with specific cell expression. The bottom panel showed velocity length and confidence of T and NK cells, with the former measuring the rate of cell differentiation and the latter measuring velocity vector correlation between neighboring cells. (**e**) t-SNE plot of T and NK cells, with each color representing different sample types and cell counts indicated in parentheses.

**Supplementary Figure 5.**

**a b**

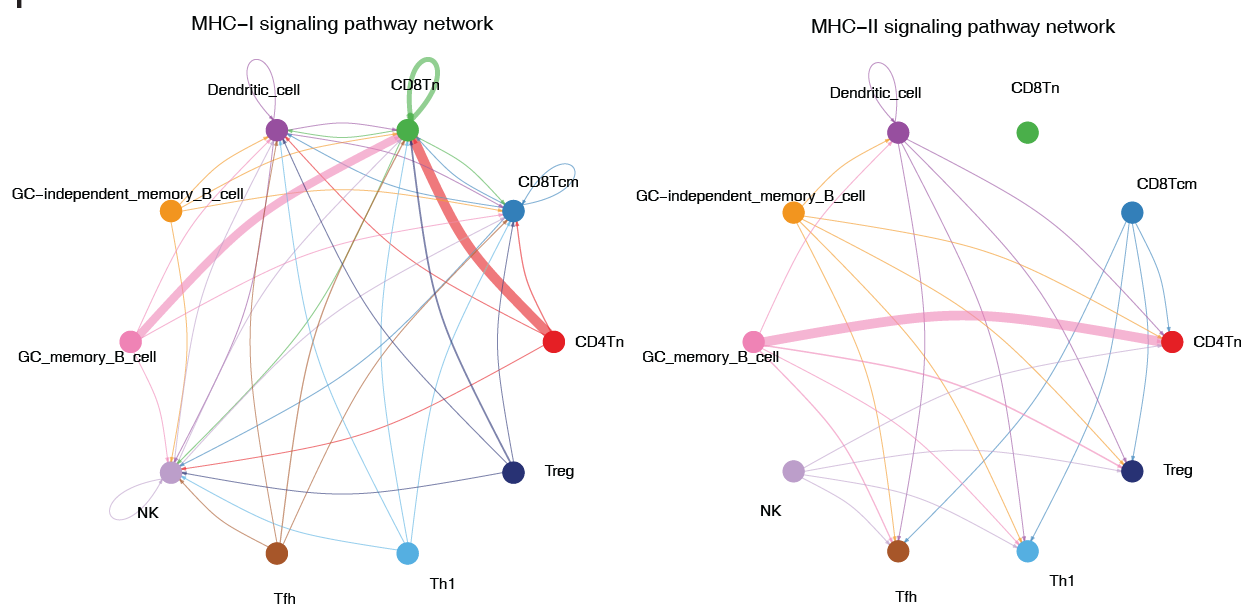


**c**

**Supplementary Figure 5. Characterizing cell-cell interactions within TLS.**

(**a**) Network diagram exhibits inferred intercellular communications among immune cells. (**b**) Bar graphs depict relative contribution of cell communication through the interaction between MHC and CD8/CD4 molecules. (**c**) Network diagrams showed inferred intercellular communications among immune cells relevant to the MHC-I and MHC-II signaling pathways.

**Supplementary Figure 6.**

**a**


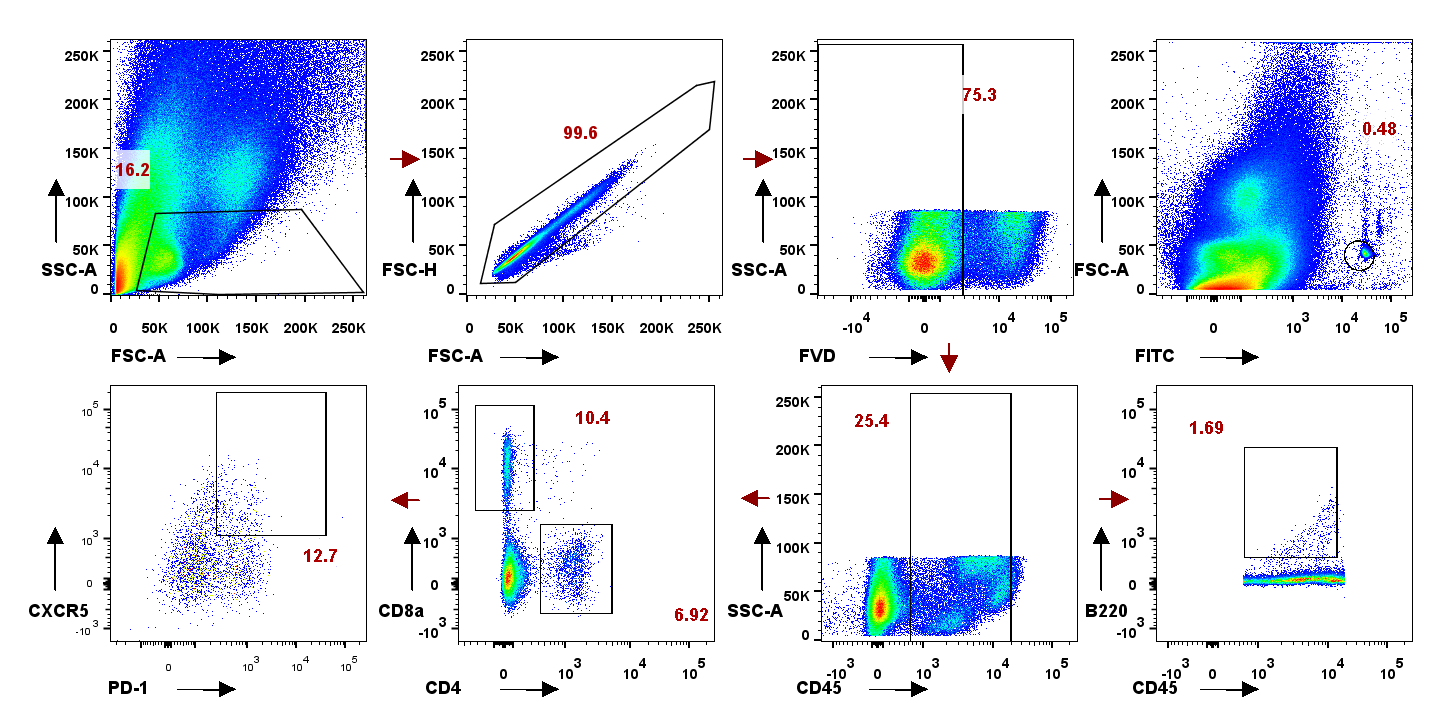


**b**


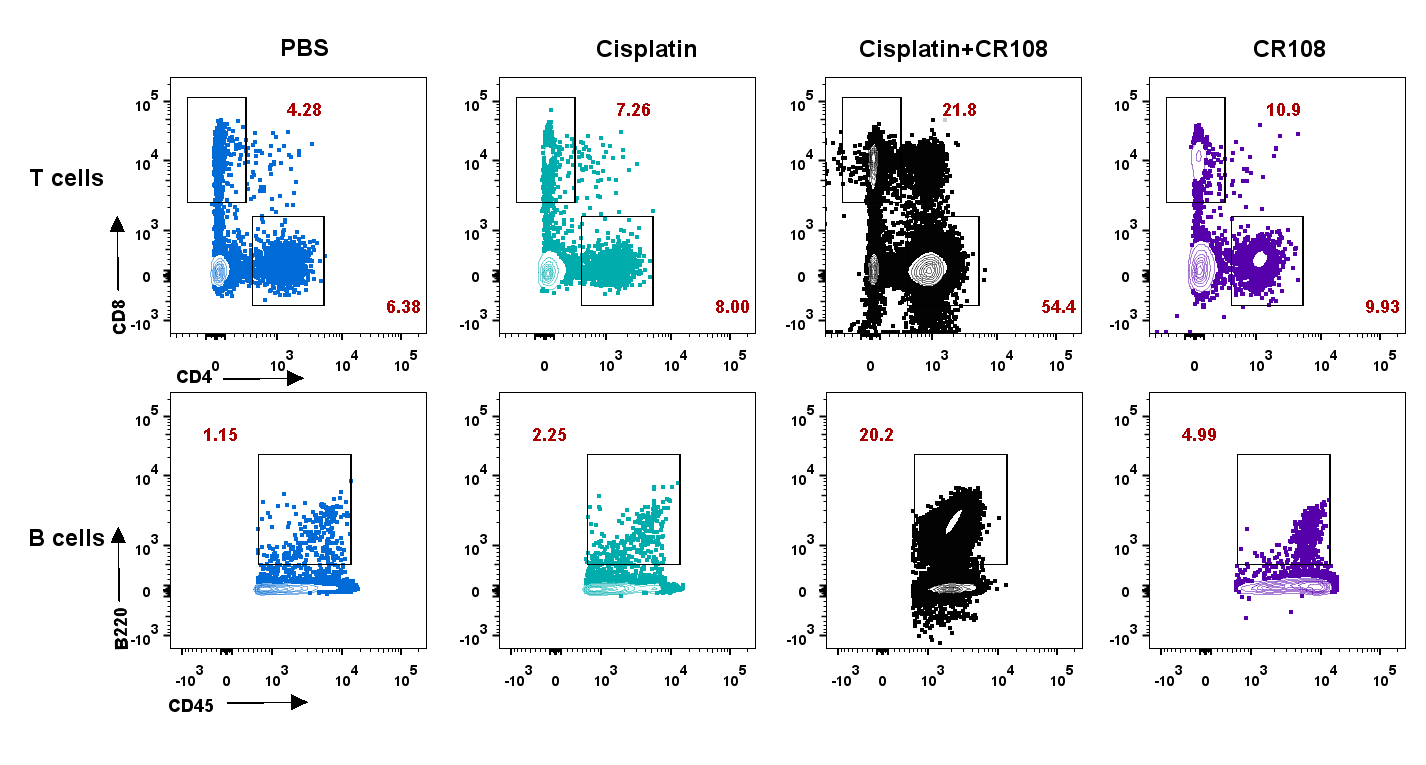


**c**


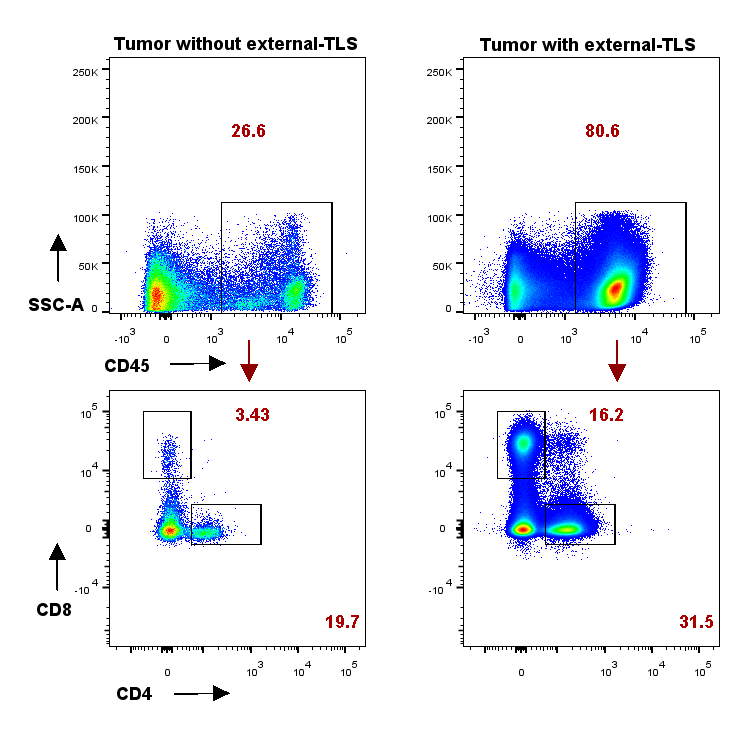


**d**


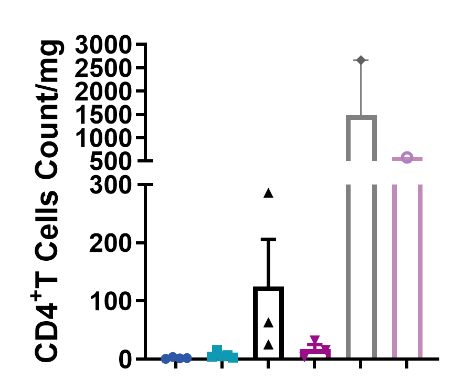

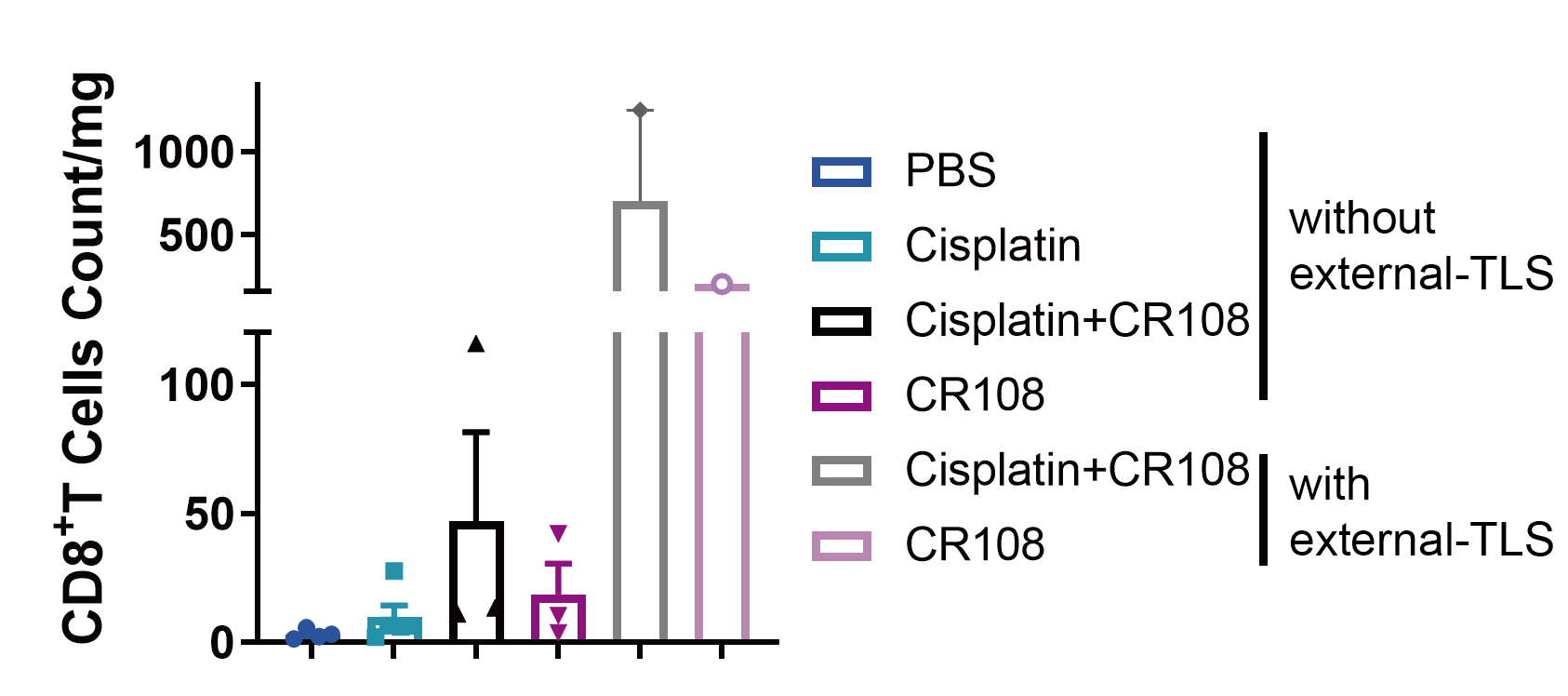

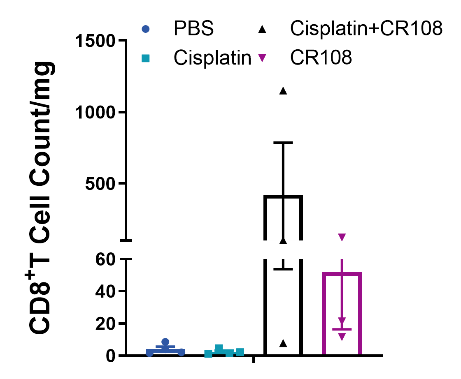


**e**


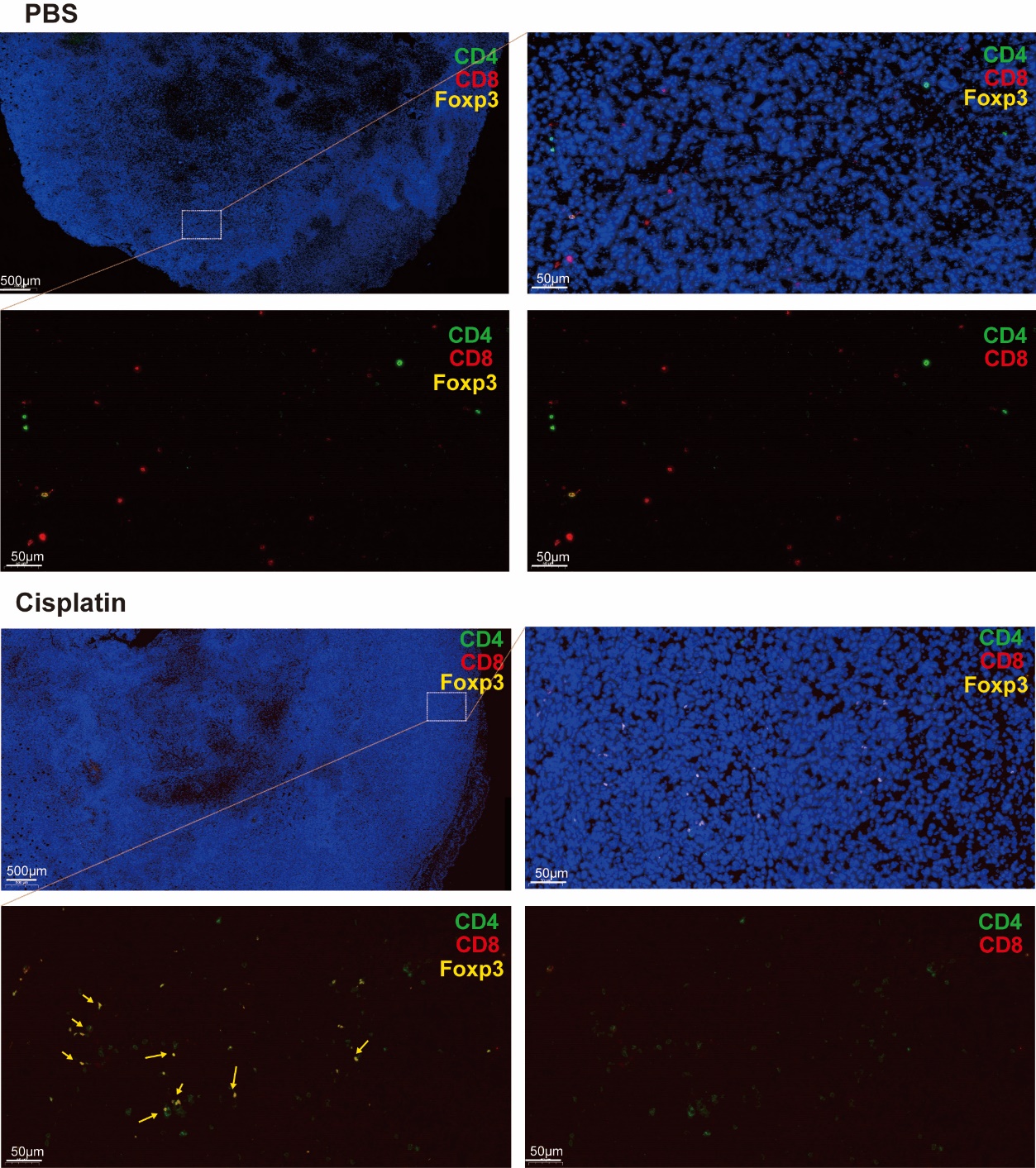


**
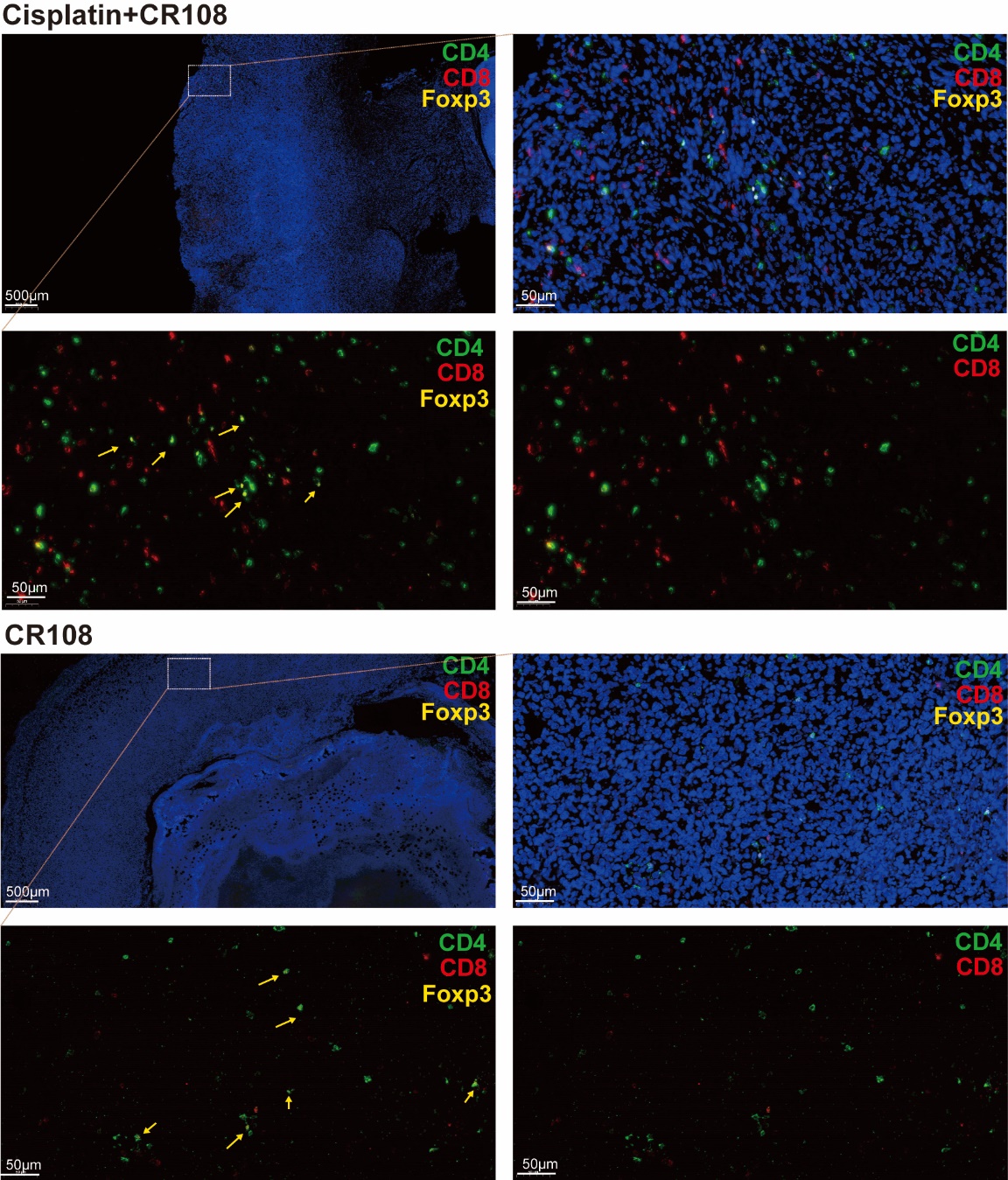
**


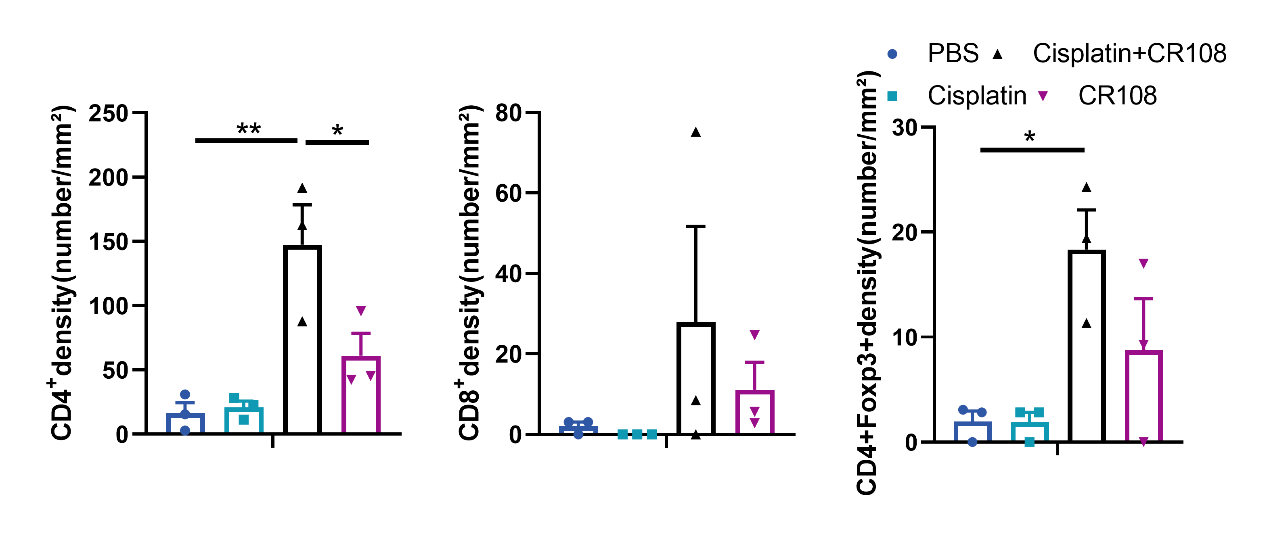


**f**

**Supplementary** **Figure 6. Quantification analysis of TILs.**

(**a**) Gating strategy of CD4^+^ T, CD8^+^ T, Tfh and B cells in TILs. (**b**) Flow cytometry plots show different amount of CD4^+^T, CD8^+^T, Tfh and B cells among PBS-treated group, cisplatin treated group, cisplatin+CR108 combo-treated group and CR108 mono-treated group in TILs. (**c**) Flow cytometry plots show different amount of CD4^+^ T and CD8^+^ T cells in tumor with external-TLS and tumor without external-TLS. (**d**) One representative analysis of two independent experiments of quantification analysis in tumor by FACS was presented. (**e**) Immunofluorescence revealed distribution of T, B cells, and Treg cells in 28 DPI tumor tissue. Fluorescence channels and corresponding targets are marked in the upper right corner of the image. The green, red, and yellow fluorescence channels represent staining for CD4, CD8, and Foxp3, respectively. Tregs are indicated by arrows. (**f**) Quantitative analysis of tumor-infiltrating CD4^+^, CD8^+^ and CD4^+^Foxp3^+^ T cells in the IF-stained specimens by counting positive cells across 3 fields of view for each biological replicate of a group. The results are expressed as mean number of positive cells per mm^2^ tissue section. Statistics: One-way ANOVA were used to determine statistical significance. Error bars: mean ± SEM.

**Supplementary Figure 7.**

**b**


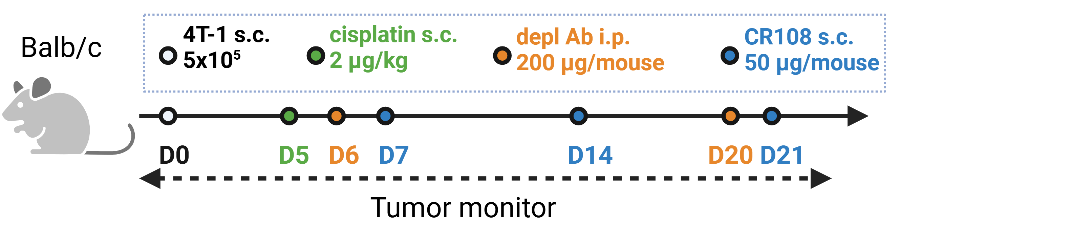


**a**

**c**

**Supplementary Figure 7. Effects of T cells mediated response in cisplatin+CR108 combo therapy on antitumor activity.**

(**a**) Schematic diagram illustrating the design of the unilateral 4T-1 tumor model with T cell depletion using antibodies (200μg/mouse CD4mAb or 200μg/mouse CD8mAb), where the dark solid dots is for the tumor cell inoculation, the green solid dot for cisplatin treatment, blue solid dot for CR108 treatments, and the orange solid dots for antibody treatments. The image was created with [www.Biorender.com](http://www.Biorender.com) and was licensed. (**b**) Assessment of depletions efficiency of antibody-mediated CD4^+^ and CD8^+^ T cell in peripheral blood, n=3. (**c**) Survival of mice after T cell depletion induced by administration of anti-mouse CD4 and anti-mouse CD8 antibodies, isotype IgG (iso) as a control, n=6.

**Supplementary Figure 8.**

**a**


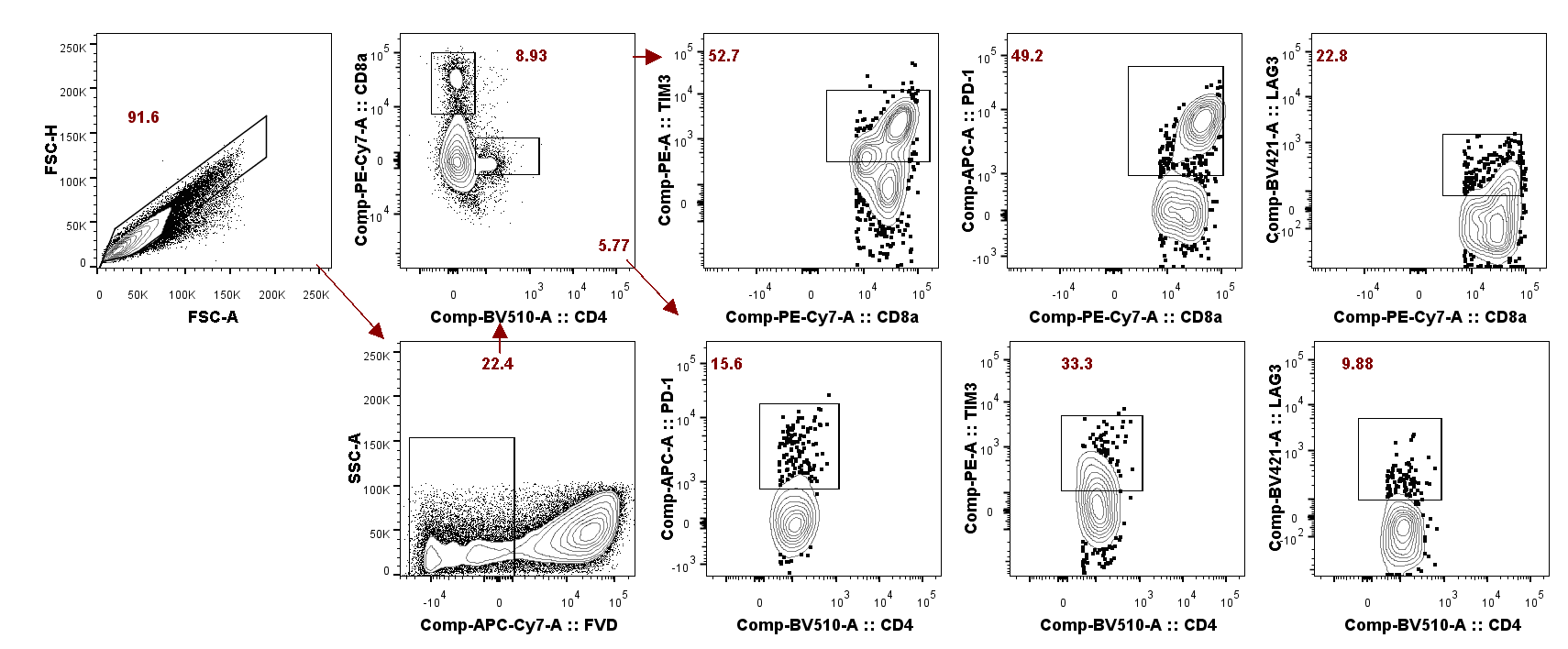


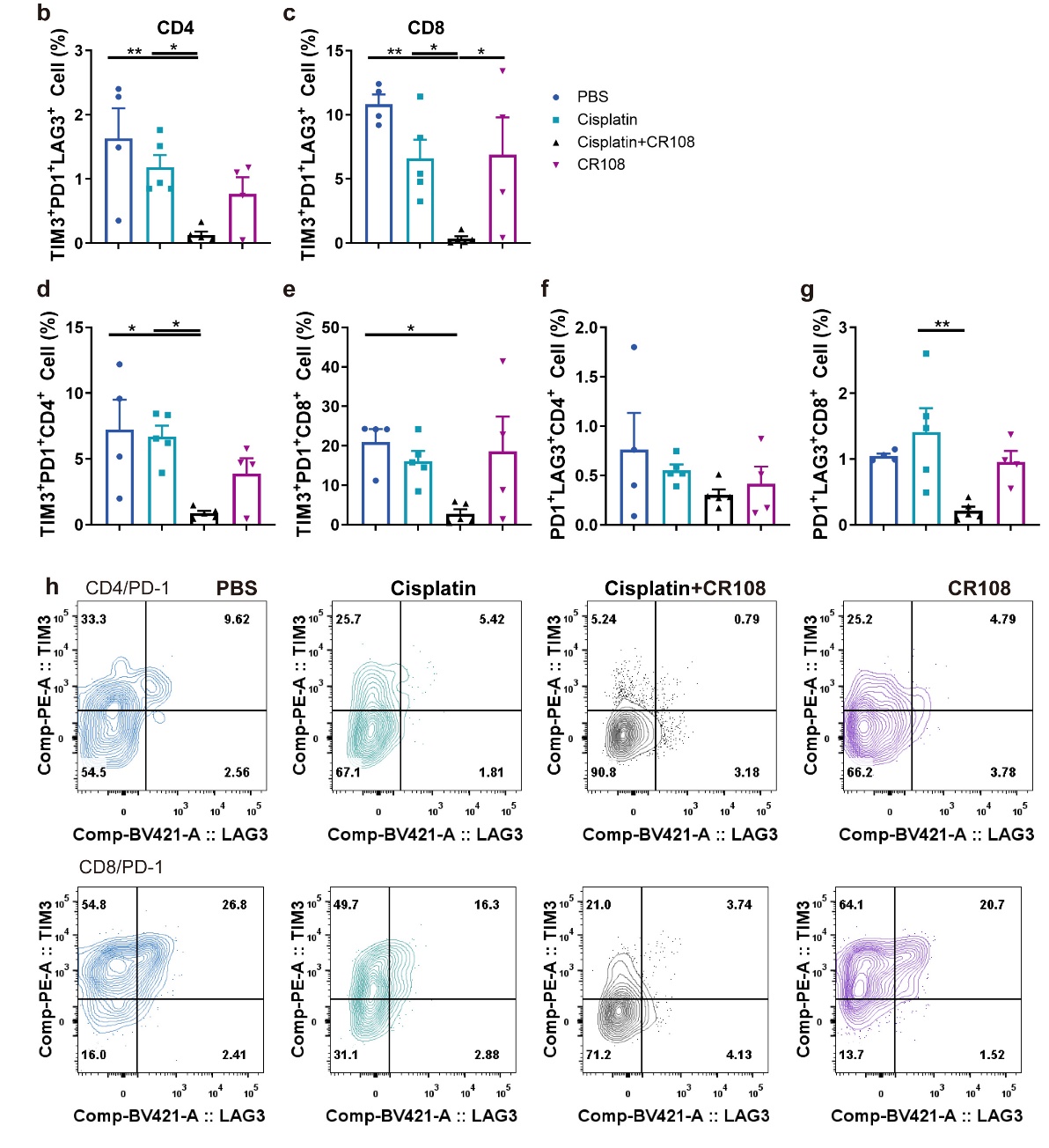


**Supplementary Figure 8.** **Functional analysis of TILs.**

(**a**) Gating strategy of exhausted marker (PD-1, TIM-3 and LAG-3) in TILs. (**b-g**) The expression of exhaustion factors of tumor-infiltrated CD4^+^ and CD8^+^T cells 28DPI was used for analysis by FACS, n=5 per group. (**h**) Flow cytometry plots show different amount of exhaustion factors in CD4^+^T, CD8^+^TILs among untreated group, cisplatin treated group, cisplatin+CR108 combo-treated group and CR108 mono-treated group. Statistics: Unmatched t test, one-way ANOVA were used to determine statistical significance. Error bars: mean ± SEM. * P < 0.05, **P < 0.001.

**Supplementary Figure S9.**

**a**


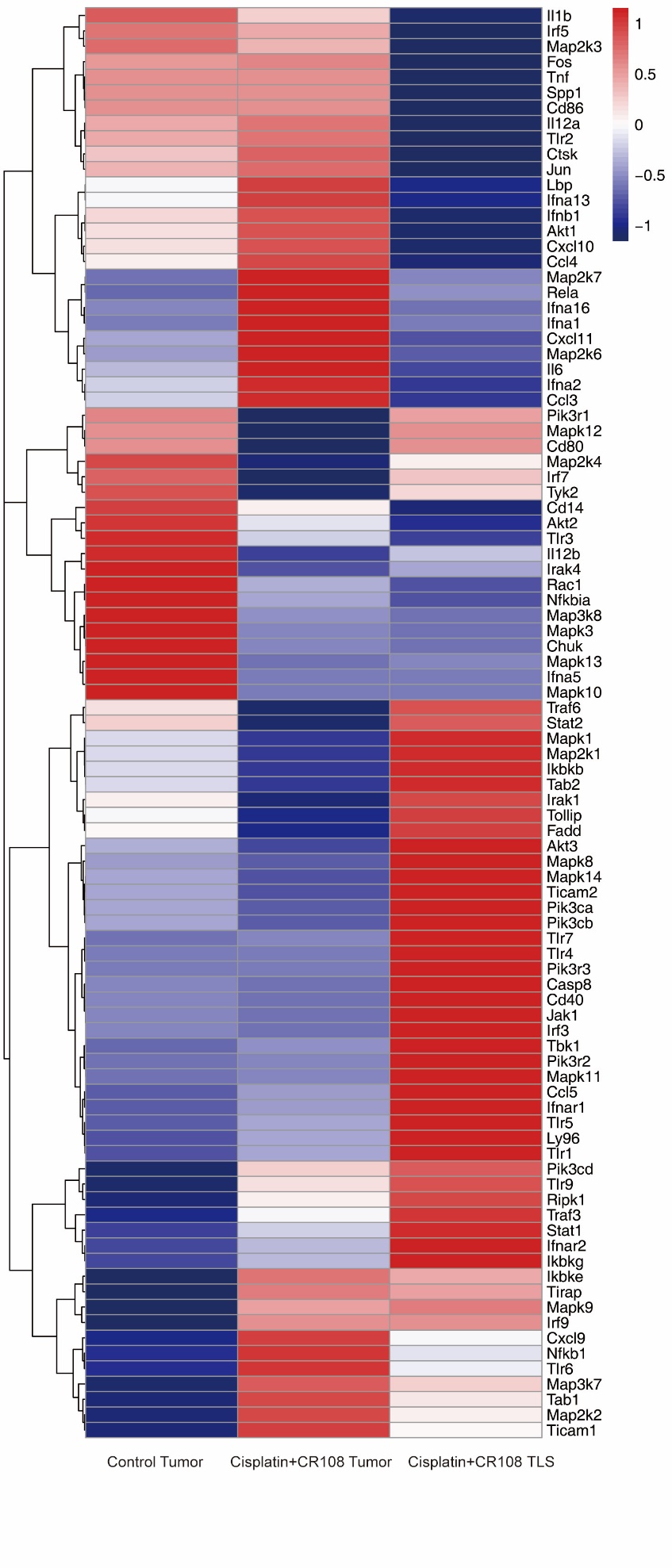


**
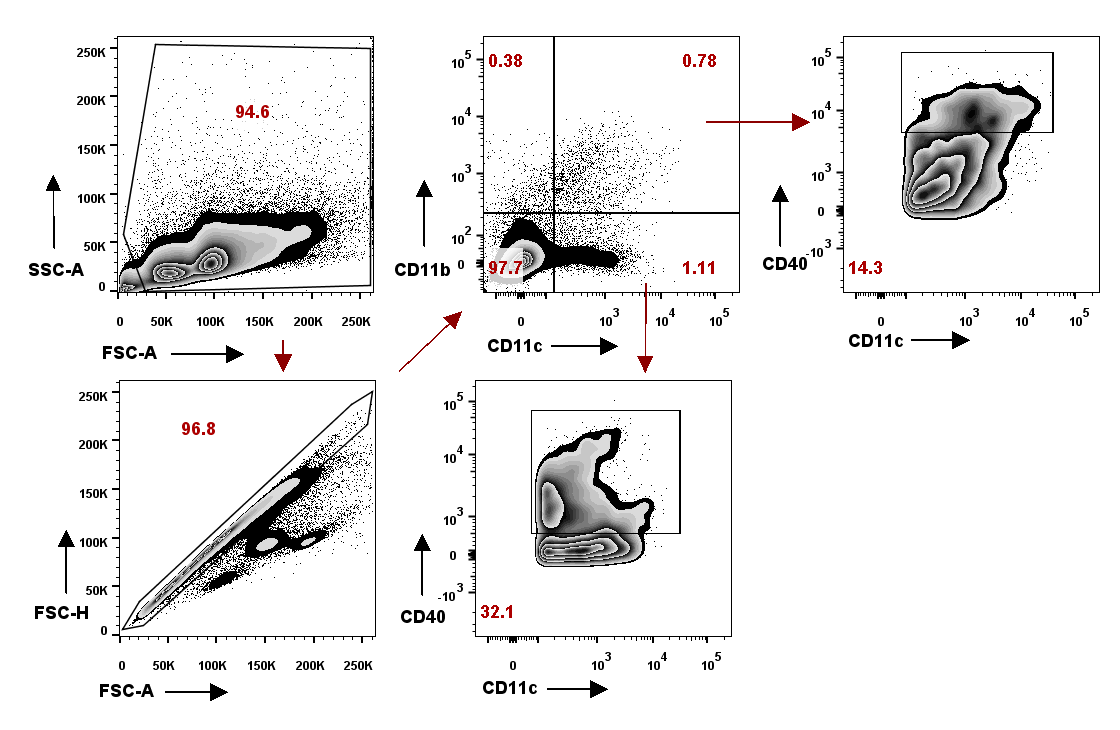
**

**b**

**
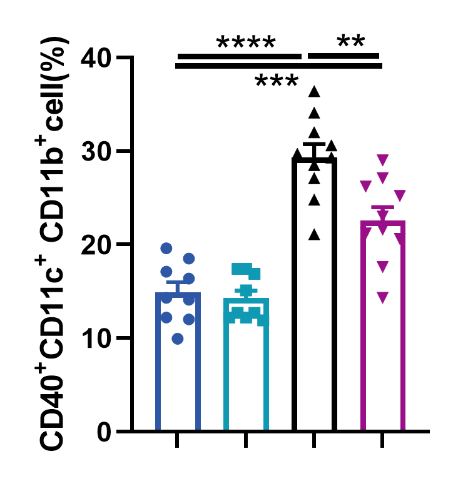

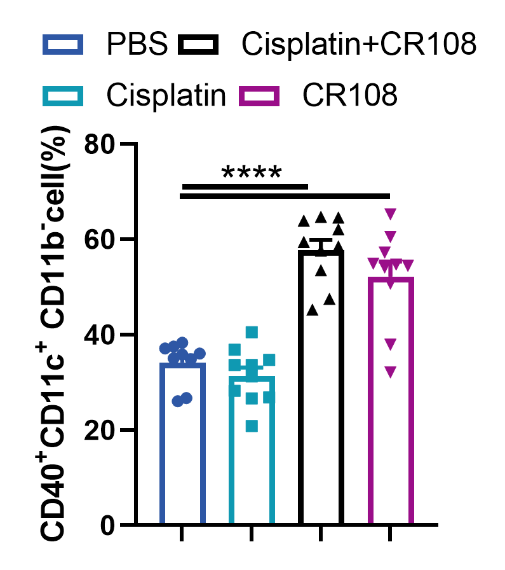
**

**c**


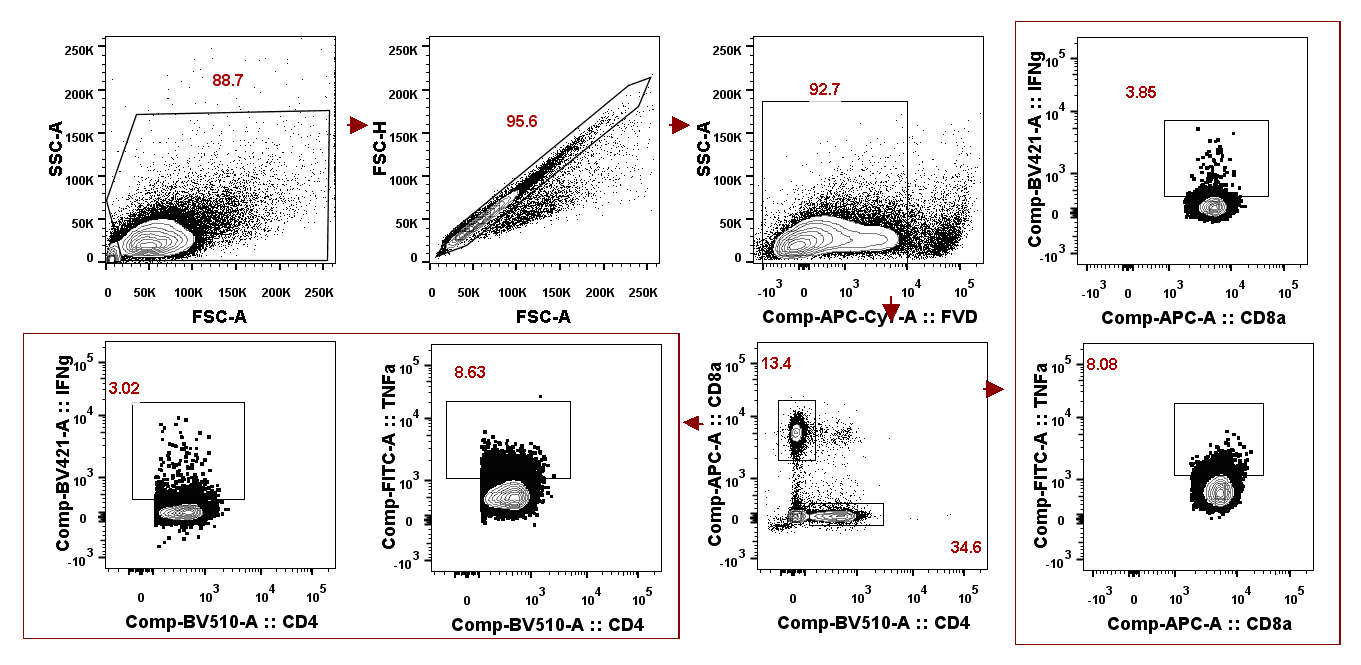


**d**

**e**


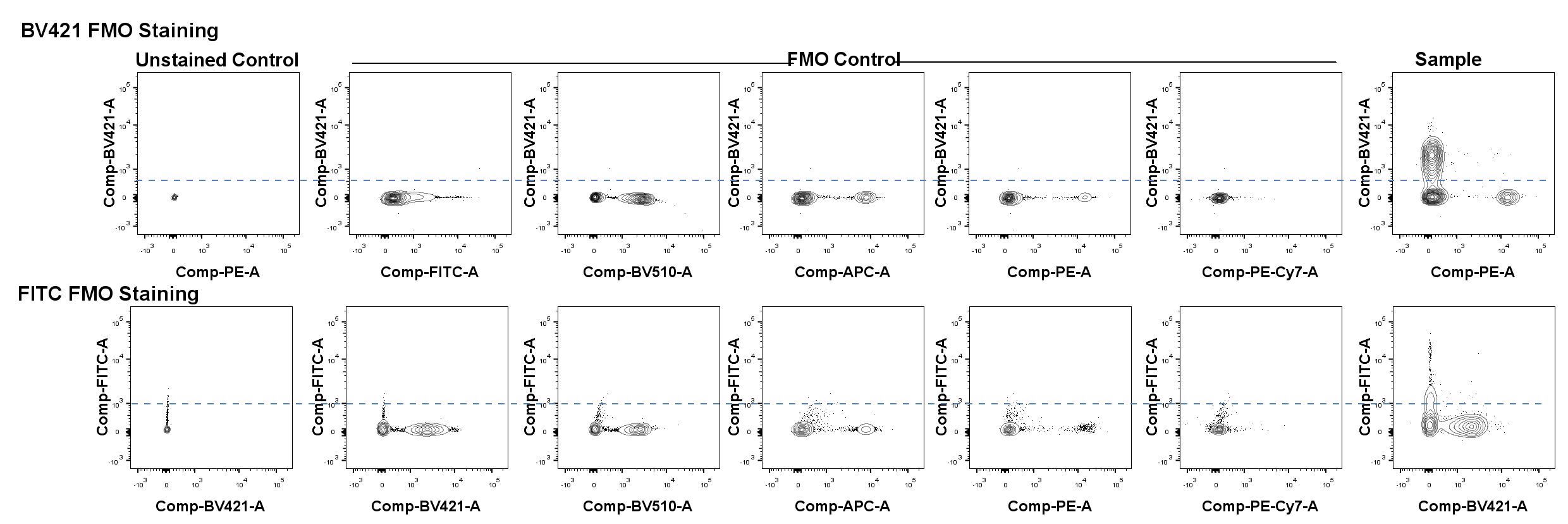


**Supplementary Figure 9.** **Assessment of TLR signal pathway in** **DCs and T cells after** **cisplatin+CR108 combo therapy.**

(**a**) Heatmap of the TLR signal pathway in PBS-treated TILs and cisplatin+CR108 combo-treated TILs. (**b**) Flow cytometric analysis of CD40 costimulatory molecule expression in DCs (both CD11c^+^CD11b^+^ and CD11c^+^CD11b^-^) from 9 DPI dLNs~~cisplatin,~~ n=10~~; CR108, n=10; others, n=10~~ (**c**) A repeat experiment of Figure 5e. One-way ANOVA was used to determine statistical significance. Error bars: mean ± SEM. **P < 0.01, ***P < 0.001. (**d**) Lymphocytes isolated from 16 DPI dLNs were stimulated with 5ng PMA and 50ng Ionomycin for 4 hrs, followed by intracellular IFN𝛾 and TNFα staining and then FACS analysis. NC, n=4; others, n=5. (**e**) Fluorescence Minus One (FMO) staining control.
